# Agonist redirected checkpoint, PD1-Fc-OX40L, for cancer immunotherapy

**DOI:** 10.1186/s40425-018-0454-3

**Published:** 2018-12-18

**Authors:** George Fromm, Suresh de Silva, Kellsey Johannes, Arpita Patel, Josiah C. Hornblower, Taylor H. Schreiber

**Affiliations:** Shattuck Labs, Inc, 21 Parmer Way, Suite 200, Durham, NC 27703 USA

**Keywords:** Cancer immunotherapy, Checkpoint, Costimulator, OX40, PD1

## Abstract

Simultaneous blockade of immune checkpoint molecules and co-stimulation of the TNF receptor superfamily (TNFRSF) is predicted to improve overall survival in human cancer. TNFRSF co-stimulation depends upon coordinated antigen recognition through the T cell receptor followed by homotrimerization of the TNFRSF, and is most effective when these functions occur simultaneously. To address this mechanism, we developed a two-sided human fusion protein incorporating the extracellular domains (ECD) of PD-1 and OX40L, adjoined by a central Fc domain, termed PD1-Fc-OX40L. The PD-1 end of the fusion protein binds PD-L1 and PD-L2 with affinities of 2.08 and 1.76 nM, respectively, and the OX40L end binds OX40 with an affinity of 246 pM. High binding affinity on both sides of the construct translated to potent stimulation of OX40 signaling and PD1:PD-L1/L2 blockade, in multiple in vitro assays, including improved potency as compared to pembrolizumab, nivolumab, tavolixizumab and combinations of those antibodies. Furthermore, when activated human T cells were co-cultured with PD-L1 positive human tumor cells, PD1-Fc-OX40L was observed to concentrate to the immune synapse, which enhanced proliferation of T cells and production of IL-2, IFNγ and TNFα, and led to efficient killing of tumor cells. The therapeutic activity of PD1-Fc-OX40L in established murine tumors was significantly superior to either PD1 blocking, OX40 agonist, or combination antibody therapy; and required CD4+ T cells for maximum response. Importantly, all agonist functions of PD1-Fc-OX40L are independent of Fc receptor cross-linking. Collectively, these data demonstrate a highly potent fusion protein that is part of a platform, capable of providing checkpoint blockade and TNFRSF costimulation in a single molecule, which uniquely localizes TNFRSF costimulation to checkpoint ligand positive tumor cells.

**Electronic supplementary material:**

The online version of this article (10.1186/s40425-018-0454-3) contains supplementary material, which is available to authorized users.

## Background

Cancer immunotherapy can be improved through combinations of individual agents, as well as through engineering biologic molecules endowed with multiple functions [1, 2]. Bi-specific antibodies are the most widely studied of this latter example, and have shown promising activity in simultaneously blocking or bridging two target molecules (PD-1, CTLA-4, CD3, CD123, etc.), with each of their two unique Fab domains [3, 4]. Similarly, engineered scFv platforms (bispecific T cell engagers, dual-affinity re-targeting, etc.) can effectively bridge two cell membrane proteins as a means to guide T cells to target antigens that are preferentially expressed by tumor cells [5, 6]. In each of these platforms, engineering high affinity binding to multiple antigens comes with the tradeoff of reduced avidity to each individual target, because whereas a native antibody binds the same epitope twice (retaining avidity), a bispecific antibody binds two epitopes once (each with a monovalent interaction, losing avidity). The loss of avidity has important consequences for activating molecules that function in vivo in dimeric or oligomeric states. This characteristic is particularly important for agonist function to receptors in the TNF superfamily, which must oligomerize to form homotrimers and hexamers for efficient signaling (i.e. OX40/OX40L, GITR/GITRL signaling, etc.) [7].

Monoclonal antibodies dominate the landscape of cancer immunotherapeutics (αPD1, αPD-L1, αCTLA4, etc.). In inflammatory disease, several Fc-containing fusion proteins including TNFRSF1b-Fc and CTLA4-Fc (etanercept and abatacept) have been highly effective in reducing inflammation in rheumatoid arthritis and psoriasis [8–11]. A number of Fc-containing fusion proteins are now in clinical development for cancer immunotherapy, including OX40L-Fc and GITRL-Fc [12, 13]. Interestingly, the common description for these fusion proteins is the same; TNFRSF1b *followed by* Fc, and OX40L *followed by* Fc, which suggests that the Fc domain is at the carboxy terminus. In reality, TNFRSF1b is a type I membrane protein with an extracellular amino terminus and OX40L is a type II membrane protein with an extracellular carboxy terminus. Thus, OX40L-Fc should correctly be referred to as *Fc-*OX40L wherein the Fc domain precedes the OX40L. The Fc region in each of these examples consists of a hinge-CH2-CH3 domain, indicating that the TNFRSF1b extracellular domain (ECD) is adjoined to the hinge domain, whereas the OX40L ECD is adjoined to the CH3 domain.

The successful development of TNFRSF1b-Fc and Fc-OX40L prompted the question of whether an ECD of a type I membrane protein could be linked through an Fc region to the ECD of a type II membrane protein in a single molecule, and importantly whether such a construct could be engineered so that each of the two functional domains would retain target binding activity. Certain pairs of membrane receptors function through intracellular signaling networks that antagonize one another. In T cells expressing PD-1, for example, engagement with PD-L1 initiates a signaling cascade involving Src homology 2-containing tyrosine phosphatase (SHP-2) which results in blockade of signaling pathways downstream of the T cell receptor and CD28 via CD3ζ and ZAP70 dephosphorylation, effectively neutralizing ‘signal 1’ in T cell activation [14–16]. There are many costimulatory receptors, including OX40 (and most other TNFRSF), which are dependent upon permissive T cell receptor signaling for activity. Specifically, OX40 does not effectively activate the PI3K/AKT pathway in the absence of T cell receptor signaling [17]. Thus, as it relates to OX40 activation, a T cell encountering antigen in the presence of PD-L1 may be a functional analogue to a T cell encountering no antigen at all. To address this biology, we developed a dual-sided Fc fusion protein platform wherein specific checkpoint and costimulatory pathways could be adjoined, collectively referred to as *Agonist Redirected Checkpoint*™ (ARC). The potentially multimeric nature of such a construct was unknown, given the fact that PD-1 typically exists as a monomer, Fc as a dimer, and OX40L as a trimer.

In this manuscript we report the development and characterization of the prototypical ARC molecule, PD1-Fc-OX40L for both human and mouse. Initial characterization confirmed the proper folding of the intact molecule through demonstrating binding to PD-L1/L2 and OX40 in both recombinant protein and cell-based assays. High affinity interaction with PD-L1/L2 and OX40 were further confirmed by surface plasmon resonance. Human PD1-Fc-OX40L (interchangeably referred to as ARC hereafter) was shown to cause activation of primary human T cells in vitro by increasing effector cytokine production and proliferation, activation of NFkB signaling in a reporter cell assay, and was also shown to outperform clinical-stage antibodies (pembrolizumab, nivolumab, tavolixizumab, and combinations of these) in stimulating IL-2 secretion in a SEB super-antigen assay. The therapeutic activity of mouse PD1-Fc-OX40L was compared to PD1 and PD-L1 blocking antibodies, OX40 agonist antibodies, or the combination of the two using in vivo models. In these studies, mouse PD1-Fc-OX40L was shown to provide higher rates of tumor rejection and long-term immunity to tumor re-challenge than any of the antibody treatments. This anti-tumor activity was primarily dependent on CD4+ T cells, and to a lesser extent, CD8+ T cells. Finally, the mechanism of action of the PD1-Fc-OX40L ARC was assessed by visualizing the functional tethering of tumor cells and T cells using immunofluorescence/time-lapse microscopy and quantitating the tumor-killing potential of these ARC stimulated T cells. Through in silico modeling, PD1-Fc-OX40L was not predicted to contain any immunogenic epitopes, which was confirmed using a large in vitro PBMC activation screen. Taken together, the PD1-Fc-OX40L ARC is able to simultaneously block immunosuppressive checkpoint signaling while also providing a costimulatory signal to T cells; thereby facilitating a potent cytotoxic anti-tumor response.

## Methods

### Antibody and recombinant protein production and purification

The coding sequences of both human and murine PD1-Fc-OX40L, and the murine PD1(K78A)-Fc-OX40L mutant [18, 19], were codon-optimized for human cell line expression and directionally cloned into pcDNA3.4-hygro-mcs expression vector (Thermo). Vectors were then transiently transfected into mammalian cells and purified using affinity chromatography. DNA sequences for nivolumab, pembrolizumab, and tavolixizumab were synthesized and cloned into pcDNA3.4 vectors, transiently expressed in CHO cells and secreted protein isolated by affinity chromatography using Protein A (GeneArt).

### Western blot

Human (h) and mouse (m) PD1-Fc-OX40L protein were treated +/− the deglycosylase PNGase F (NEB, Cat #P0704) for 1 h at 37 °C according to manufacturer’s recommendations, and then +/− the reducing agent beta-mercaptoethanol. Samples were diluted in RIPA buffer prior to separation by SDS-PAGE. Primary antibodies used for probing hPD1-Fc-OX40L and mPD1-Fc-OX40L were obtained from Cell Signaling Technology, Jackson ImmunoResearch Laboratories, Inc. and R&D Systems.

### Functional ELISA

For characterization of the h/m PD1-Fc-OX40L, high-binding ELISA plates (Corning) were coated overnight at 4 °C with 5 μg/mL of either recombinant hFc, hPD-L1-Fc, hOX40-His, mFc, mPD-L1-Fc or mOX40-His, in PBS (reagents were obtained from Jackson ImmunoResearch Laboratories, Sino Biological, Inc., Acro Biosystems and R&D Systems). Plates were then blocked with casein buffer for 1 h at room temperature (RT) and then probed with serial dilutions of the h/m PD1-Fc-OX40L, along with the appropriate standards (human and mouse; IgG, PD1-Fc, and OX40L-Fc) for 1 h at RT. Plates were washed and then detection antibody was added for 1 h at RT in the dark. Detection antibodies included anti-hIgG-HRP, anti-mIgG-HRP, anti-hOX40L and then anti-Goat-HRP, anti-mOX40L and then anti-Goat-HRP (all antibodies were obtained from Jackson ImmunoResearch Laboratories or R&D Systems). Plates were washed again and SureBlue TMB Microwell Peroxidase Substrate (KPL) was added to each well and allowed to incubate at RT for 20 min in the dark. To stop the reaction, 100 μL of 1 N sulfuric acid was added to each well and absorbance at 450 nm was read immediately on a BioTek plate reader. Samples were run at a minimum in triplicate and at multiple dilutions.

For the PD1-blocking ELISA, hPD-L1-Fc (Sino Biological, Inc.) was used to coat a high binding ELISA plate as described above. The following day, binding was detected using a recombinant hPD1-Biotin protein (Acro Biosystems) in combination with increasing concentrations of either hPD1-Fc-OX40L (to compete with PD1-Biotin binding for PD-L1) or an irrelevant fusion protein, which served as a negative control. Following this incubation, plates were washed as described above and probed with an avidin-HRP detection antibody (BioLegend) for 1 h at RT in the dark. Plates were then washed and analyzed as above. For IL2 (mouse and human) and TNFα functional ELISAs, cell culture supernatants were collected and assessed using commercial ELISA kits according to manufacturer recommendations (human IL2, human TNFα, and mouse IL2; R&D Systems).

### Surface Plasmon resonance

Direct binding of h/m PD1-Fc-OX40L fusion protein to recombinant protein targets was performed using a BioRAD ProteOn™ XPR36 protein interaction array instrument. To determine the on-rates (Ka), off-rates (Kd) and binding affinities (KD) of hPD1-Fc-OX40L to its intended binding targets (referred to as ‘Ligands’), histidine-tagged versions of the human recombinant targets - PD-L1 (Acro Biosystems), PD-L2 (Acro Biosystems), OX40 (Acro Biosystems), FcγRIA (Sino Biological Inc.), FcγRIIB (Sino Biological Inc.), FcγRIIIB (Sino Biological Inc.) and FcRn (R&D Systems) were immobilized to an Ni-sulfate activated ProteOn™ HTG sensor chip (BioRAD). Increasing concentrations of the hPD1-Fc-OX40L fusion protein (referred to as ‘Analyte’) diluted in PBS/Tween (0.005%) buffer pH 7.0 was injected for 3 min followed by a dissociation phase of 5 min at a flow rate of ≥80 μl/min. hPD1-Fc and hOX40L-Fc (Acro Biosystems) recombinant proteins were used as positive control analytes for binding to their respective partners (PD-L1/L2 and OX40, respectively) and human IgG was used as a positive control for binding to Fcγ receptors and FcRn. To assess analyte binding to FcRn ligand, the pH of the buffer was reduced to pH 5.5. To minimize mass transport limitation, ligand concentrations were maintained at an optimized low concentration and flow rates over the chip were maintained at 80–100 μL/min.

Mouse PD1-Fc-OX40L fusion protein binding to mouse counterparts of the same targets was assessed as described above and the relevant recombinant proteins were obtained from Acro Biosystems, and R&D Systems.

### Cell culture

Jurkat, CHO-K1, HeLa, HCC827, PC3, NCI-H2023, 4 T1, B16.F10, and CT26 cells were maintained in IMDM media supplemented with glutamine and 10% fetal bovine serum (FBS) at 37 °C in 5% CO_2_.

### In vitro cell line generation

Stable cells lines were generated to assess in vitro binding of human and mouse PD1-Fc-OX40L ARC proteins (CHO-K1/hPD-L1, CHO-K1/hPD-L2, Jurkat/hOX40, HeLa/hOX40, CHO-K1/mPD-L1, CHO-K1/mPD-L2, and CHO-K1/mOX40). Briefly, cDNAs were amplified from CD3/CD28 activated human PBMCs or mouse splenocytes, and cloned into pcDNA3.1(−) (Life Technologies). Parental CHO-K1 and Jurkat cells were nucleofected with the 4D-Nucleofector and Cell Line Nucleofector Kit SE (Lonza) according to manufacturer’s directions. Cells were selected with antibiotics and single-cell cloned in order to generate stable, high-level expressing clones that were screened by flow cytometry.

### Tumor model systems

For CT26 studies, BALB/C mice were subcutaneously implanted with 5 × 10^5^ tumor cells into the rear flank on day 0. On treatment days, tumor bearing mice were either untreated or treated with 2 doses (on days 5 and 7) of the mPD1-Fc-OX40L ARC, anti-OX40 (clone OX86; BioXcell), anti-PD1 (clone RMP1–14; BioXcell), or anti-PD-L1 (clone 10F.9G2; BioXcell). Tumor area (mm^2^) and overall survival was assessed throughout the time-course. Survival criteria included total tumor area less than 175 mm^2^ with no sign of tumor ulceration. Complete responders, in which tumors established and were subsequently rejected are listed in the appropriate figures. A cohort of CT26 experimental mice was euthanized on day 13 for immune profiling in splenocytes and tumor tissue using flow cytometry. Serum was isolated from peripheral blood and cytokine levels were assessed using LEGENDplex (BioLegend) cytokine reagents. Tumors were excised from these mice and dissociated using a tumor dissociation kit (Milltenyi) and homogenized through a 100 μM strainer to isolate tumor cells and infiltrating immune cells.

For CD4/CD8 depletion experiments, mice were treated via IP injection of 100 μg of CD4 (clone GK1.5, BioXcell), CD8 (clone 2.43, BioXcell), or both CD4/CD8 depleting antibodies on days − 1, 1, and 10 of the experimental time-course. CT26 tumors were inoculated on day 0, as described above. Vehicle (PBS) or mPD1-Fc-OX40L (300 μg) treatments were given via IP injection on days 5 and 7 of the time-course. CD4 and CD8 populations in the peripheral blood were assessed at several time points to verify depletion.

### LEGENDplex cytokine analysis

Experimental mice were euthanized through CO_2_ asphyxiation and cervical dislocation and whole blood was collected via cardiac puncture. Blood was allowed to coagulate for 30 min at room temperature and spun at 1000 g for 10 min to separate the serum. Serum was then transferred to a new 1.5 mL eppendorf tube. Cytokine analysis was performed using the LEGENDplex Cytokine Analysis kit (BioLegend) according to manufacturer recommendations and analyzed on the Sony SH800 or BD LSRII Fortessa. Flow cytometry results were converted to pg/mL secretion using BioLegend LEGENDplex software.

### Flow cytometry

Briefly, isolated cells were stained with antibodies for 30 min on ice in the dark. Indicated antibodies were purchased from either Sony, BioLegend, or Abcam. Following this incubation period, stained cells were washed and resuspended in FACS buffer (1X PBS buffer containing 1% bovine serum albumin (BSA), 0.02% sodium azide and 2 mM EDTA). The AH1 tetramer (SPSYVYHQF) was purchased from MBL International. For intracellular FOXP3 staining, cells were fixed and permeabilized using the True-Nuclear Transcription Factor Buffer Set from Biolegend. Flow cytometry and cell sorting were performed on a Sony SH800 or BD LSRII Fortessa.

### In vitro functional assays

#### Human co-culture assay

CD3+ T cells were isolated from PBMCs using a magnetic selection kit (Stemcell Technologies), and then stimulated for 2 days with a suboptimal quantity of bead bound CD3/CD28 (ThermoFisher) and soluble IL2 (BioXcell). During these 2 days, PC3 (PD-L1^Low^) and HCC827 (PD-L1^High^) cells were plated in multi-well tissue culture coated plates. After these 2 days, PC3 and HCC827 cells were treated with Mitomycin-C (Sigma) for 2 h 37 °C and 5% CO_2_ in order to prevent further cell division. Activated T cells were then plated on the Mitomycin-C treated tumor cells +/− PD1-Fc-OX40L for 5 additional days. On day 6 after initial T cell isolation, supernatants were collected from co-cultures and assessed using IL2 ELISA.An initial time-course was performed and determined that maximal proliferation and cytokine responses were observed between days 5 and 7 of the co-culture (data not shown). Thus, on days 5 and 7 after initial T cell isolation, the T cells in suspension in the co-culture systems were isolated and analyzed by flow cytometry for markers associated with proliferation (Ki-67) and T cell activation (IFNγ and TNFα).

The mouse functional co-culture assay depicted in Additional file 1: Figure S5H-I was performed identical to the human experiment performed above, however B16.F10 (PD-L1^High^) and 4 T1 (PD-L1^Low^) cells were used, as well as a mouse CD3 isolation kit (Stemcell Technologies), mouse CD3/CD28 beads (ThermoFisher), mouse IL2 (BioXcell), and a mouse IL2 detection ELISA.

#### Staphylococcus enterotoxin-B (SEB) assay

Primary PBMCs (or mouse splenocytes) were obtained from healthy donor leukopacs (Stemcell technologies) and incubated +/− increasing concentrations of the super-antigen SEB (List Biological Laboratories), in the presence or absence of the PD1-Fc-OX40L ARC, human/mouse IgG (Jackson Immunoresearch, Inc.), antibody controls, or single-sided fusion protein controls (Acro Biosystems and Sino Biologicals); as both monotherapies and combinations. All antibodies and ARC proteins were used at the concentrations indicated in the figures. After 3 days, culture supernatants were collected and assessed by ELISA for levels of IL-2 (R&D Systems). Additionally, the SEB assay was performed in human PBMCs depleted of CD4, CD8, or both CD4/CD8 cells, using magnetic positive selection kits (StemCell Technologies).

To account for variability between donors when directly comparing PD1-Fc-OX40L activity to antibody/fusion protein comparators, OD450 values were baseline-normalized by setting the first IgG control OD450 value to .1, and subtracting each treatment group by the numerical value needed to obtain the .1 control value. The concentration of secreted IL-2 was also quantitated in certain experiments, and ranged between ~ 500–2000 pg/mL.

#### Proliferation assay

Primary PBMCs were isolated and incubated as described above for the SEB assay. After 3 days, when the culture supernatant was removed for IL-2 ELISA analysis, proliferation of the remaining cells in culture was assessed using the CellTiter 96 Aqueous One Solution Cell Proliferation Assay (MTS) (Promega) according to manufacturer’s instructions, and quantitated on a GloMax Navigator luminometer (Promega).

#### NFkB-luciferase reporter assay

The in-house engineered Jurkat/hOX40 cells, were stably transfected to generate a stable cell line that co-expressed an NFkB-luciferase reporter plasmid, pGL4.10[luc2] (Promega). Bright-Glo luciferase reagent and a luminometer were used to assess activation of NFkB signaling. The expression vector, luciferase reagent, and luminometer (Promega) were used according to manufacturer guidelines. Briefly, anti-CD3 (OKT3, at 1 μg/mL) was diluted in PBS and used to coat white 96-well plates (Costar) over night at 4 °C. The next morning, plates were washed to remove unbound anti-CD3 and 10,000 Jurkat/hOX40/NFkB-luciferase cells were plated in each well with 2 μg/mL functional grade CD28 (eBiosciences), +/− the ARC or other test agents. Plates were incubated at 37 °C/5% CO_2_ for 6 h, where after the Bright-Glo reagent was added and luminescence was assessed on a luminometer.

#### Immunofluorescence

Human CD3+ cells were isolated and stimulated as described above, for 2 days. The day before co-culture, NCI-H2023 tumor cells were plated in 96-well 190 μM glass bottom plates (Greiner). The following day, tumor cells were treated for 30 min with Cell Tracker Orange (Invitrogen), HCS Nuclear Mask Blue (Invitrogen), and for 1 h with 150 μg of the human PD1-Fc-OX40L ARC previously labeled with Alex Fluor 647 (Invitrogen). Media was then removed and activated human T cells were seeded on the tumor cells at a ratio of 8:1 (T cell: tumor cell), along with CellEvent Caspase 3/7 Green Detection Reagent (Invitrogen). Images were then acquired over the course of 6 h at a 40X magnification on the GE IN Cell Analyze 2500HS System.

#### CD107a degranulation assay

C57Bl/6 mice were adoptively transferred with 5 × 10^5^ OT-I (GFP) and 1 × 10^6^ OT-II cells via tail vein injections. The following day, mice were vaccinated with Ova/Alum prepared as previously described [20]. Three days after vaccination, CD3+ splenocytes were harvested and activated for 2 days according to the procedure described above. The day before co-culture, B16.F10-ova cells were plated in 24 well tissue culture plates. The following day, T cells were seeded on top of tumor cells at a ratio of 8:1 (T cell: tumor cell) +/− test agents. After 24 h, brefeldin A (BFA) and GolgiStop (BD Biosciences) were added to the cultures for 4 h and returned to the tissue culture incubator. After 4 h, suspension cells were removed and stained for surface expression of CD4, Vβ5.1/5.2, CD8, and CD107a, and intracellular expression of IFNγ (according to the flow cytometry procedure described above), and then analyzed by flow cytometry. All antibodies were purchased from either BioLegend or BD Biosciences.

#### Cleaved caspase 3/7 luciferase assay

The co-culture setup for this assay was identical to the CD107a assay above. Six hours after co-culture in white 96-well plates, apoptosis was assessed by reading out cleaved caspase 3/7, using the Caspase-Glo 3/7 Assay from Promega, according to manufacturer recommendations.

#### TUNEL assay

The co-culture setup for this assay was identical to the CD107a assay above. 1–3 days after co-culture in 96-well plates, DNA fragmentation was assessed using the TiterTACS Colorimetric Apoptosis Detection (TUNEL) Kit from Trevigen, according to manufacturer recommendations.

#### Immunogenicity analysis

##### Proliferation

PBMCs were isolated from 50 healthy donor buffy coats and CD8+ T cells were depleted using CD8+ RosetteSep (StemCell Technologies). Cells were plated at a density of 4–6 million cells per mL in AIM-V medium (Gibco). On days 5, 6, and 7, cells were gently resuspended in 3 × 100 μL aliquots and transferred to each well of a round bottomed 96 well plate. Cultures were pulsed with 0.75 μCi [^3^H]-Thymidine (Perkin Elmer) in 100 μL AIM-V culture medium, and incubated for a further 18 h, before harvesting onto filter mats (Perkin Elmer), using a TomTec Mach III cell harvester. Counts per minute (Cpm) for each well were determined by Meltilex (Perkin Elmer) scintillation counting on a 1450 Microbeta Wallac Trilux Liquid Scintillation Counter (Perkin Elmer) in paralux, with low background counting.

##### IL-2 ELISpot

PBMCs were isolated, CD8 cells depleted, and cultured in AIM-V as described above, and on day 8, cells were assessed. Briefly, ELISpot plates (Millipore) were pre-wetted and coated overnight with 100 μL/well IL-2 capture antibody (R&D Systems) in PBS. Cells were plated at a density of 4–6 million cells/mL in a volume of 100 μL per well, in sextuplicate. After 8 days, ELISpot plates were developed by sequential washing in dH_2_O and PBS (× 3), prior to the addition of 100 μL filtered biotinylated detection antibody (R&D Systems). Following incubation at 37 °C for 1.5 h, plates were further washed and incubated with 100 μL filtered streptavidin-AP (R&D Systems) for 1 h; and then washed again. 100 μL BCIP/NBT substrate (R&D Systems) was added to each well for 30 min at room temperature. Spot development was stopped by washing wells 3 times with dH_2_O. Dried plates were scanned on an Immunoscan Analyser and spots per well (SPW) were determined using Immunoscan Version 5 software.

Samples included human PD1-Fc-OX40L (.3 μM), the neoantigen KLH (Keyhole limpet haemocyanin; .3 μM) as a positive control, and Exenatide (Bydureon, 20 μM) as a clinical benchmark control; representing a non-immunogenic agent in this assay. For ELISpot, a mitogen positive control (PHA at 8 μg/mL) was included on each plate as an internal test for ELISpot function and cell viability.

An empirical threshold or Stimulation Index (SI), equal to or greater than 1.9 has been previously established (by the Contract Research Organization Abzena) whereby samples inducing responses above this threshold are deemed positive. SIs were calculated for each test article by dividing each value by the mean of media only control wells. One-way ANOVA was used to calculate significance versus the Exenatide control.

### Experimental animal guidelines

All mouse protocols were designed based on IACUC guidelines and approved by a licensed veterinarian. Experimental mice were monitored daily and euthanized by CO_2_ asphyxiation and cervical dislocation prior to any signs of distress.

### Statistical analysis

Experimental replicates (N) are shown in figures and figure legends. Unless noted otherwise, values plotted represent the mean from a minimum of 3 distinct experiments and error is SEM. Statistical significance (*p*-value) was determined using One-Way ANOVA with multiple comparisons. Significant *p*-values are labeled with one or more ‘*’, denoting *p < .05, **p < .01, ***p < .001 and ****p < .0001. Mantel-Cox statistical tests were used to determine the significance between the survival curves shown in Figs. 5 and 6. *P*-values are noted in the legends to these figures.

## Results

### Production and characterization of PD1-fc-OX40L

**Fig. 1:**
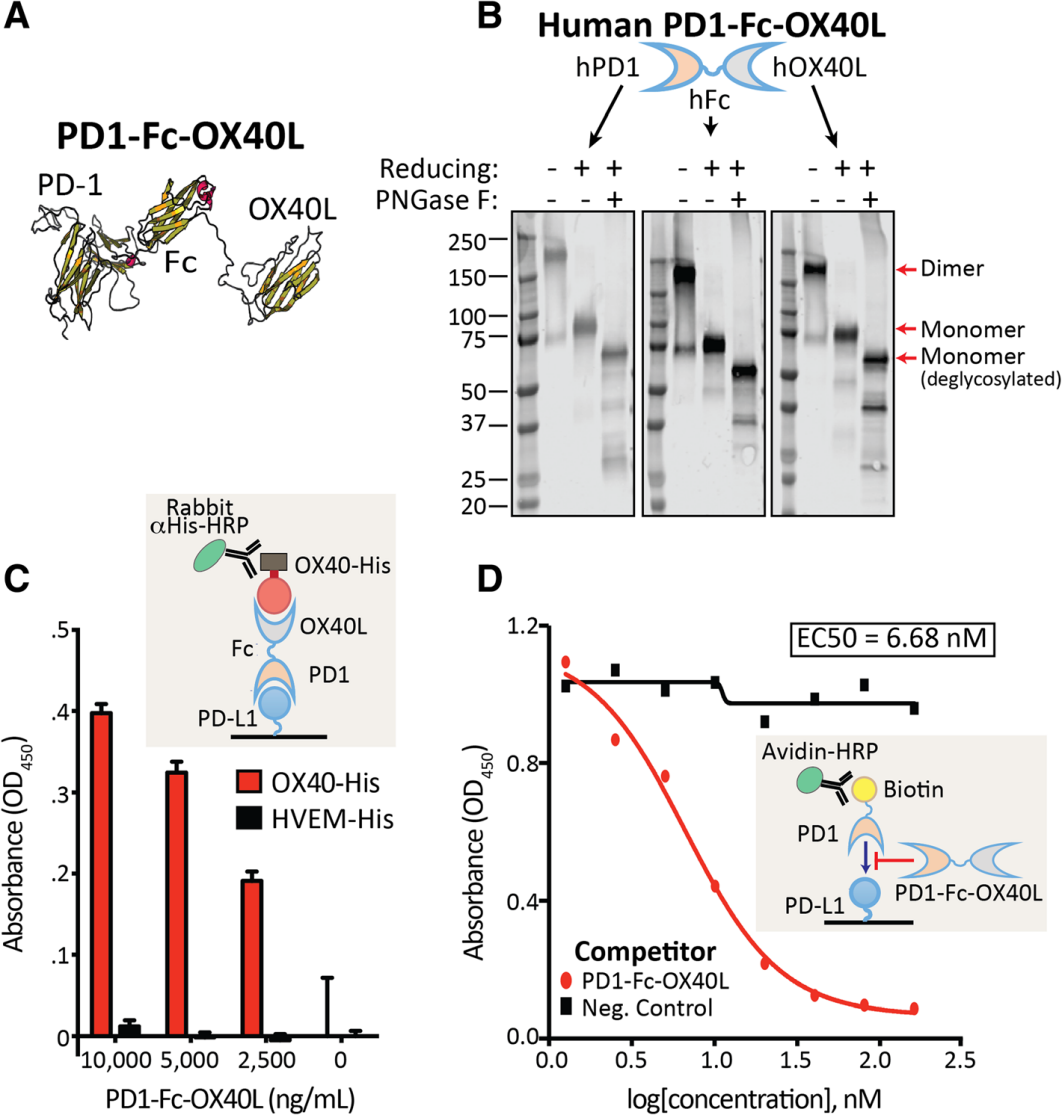
PD1-Fc-OX40L ARC retains proper folding and binding to PD-L1 and OX40. **a** Predicted tertiary structure of human PD1-Fc-OX40L. **b** Western blot analysis of PD1-Fc-OX40L performed by probing purified protein with human anti-PD1, anti-Fc, and anti-OX40L, under non-reducing and reducing conditions, and ± the deglycosylase PNGase F. **c** Functional ELISA using capture with recombinant hPD-L1 followed by detection with recombinant hOX40-His and then anti-His-HRP. HVEM-His serves as a negative control. **d** Functional PD-L1 blocking assay testing the ability of hPD1-Fc-OX40L to outcompete PD1-Biotin for binding to plate bound PD-L1 in an ELISA format. Avidin-HRP was used for signal detection

To generate the first ARC construct, the ECDs of PD1 and OX40L were fused via an antibody Fc domain for both human and mouse to generate PD1_ECD_-Fc-OX40L_ECD_; hereafter referred to as PD1-Fc-OX40L. In silico structural modeling predicted that each individual domain of the adjoined construct would fold in accordance with the native molecules, which would ensure preservation of both binding functions (Fig. 1a and schematic in Additional file 2: Figure S1A-C). Chinese hamster ovary (CHO) cells were then transfected with the PD1-Fc-OX40L expressing construct and the secreted protein was purified from conditioned media by affinity chromatography. A single peak of protein was eluted from these columns, and the presence of the individual domains within the fusion protein was confirmed by Western blotting by anti-PD1, anti-Fc and anti-OX40L antibodies (Fig. 1b). These blots revealed a glycosylated protein that forms a dimer under non-reducing conditions by SDS-PAGE. The reduced and deglycosylated form of the protein migrated at the predicted monomeric molecular weight of 57.6 kDa. To determine whether PD1-Fc-OX40L retained binding to both PD-L1 and OX40, functional ELISA assays were developed to quantify and demonstrate simultaneous binding of PD1 to recombinant PD-L1 and OX40L to recombinant OX40 (Fig. 1c). Individual ELISAs confirmed binding to recombinant human Fc and OX40 (Additional file 3: Figure S2A-B). Additionally, functional activity and ability to outcompete/block a recombinant human PD1-Biotin protein was assessed in a separate ELISA based assay (Fig. 1d). Human PD1-Fc-OX40L efficiently out-competed a commercially available recombinant human PD1-biotin for binding to plate bound PD-L1, generating an EC50 of 6.68 nM.

### PD1-fc-OX40L binds with high affinity to cognate targets

**Fig. 2:**
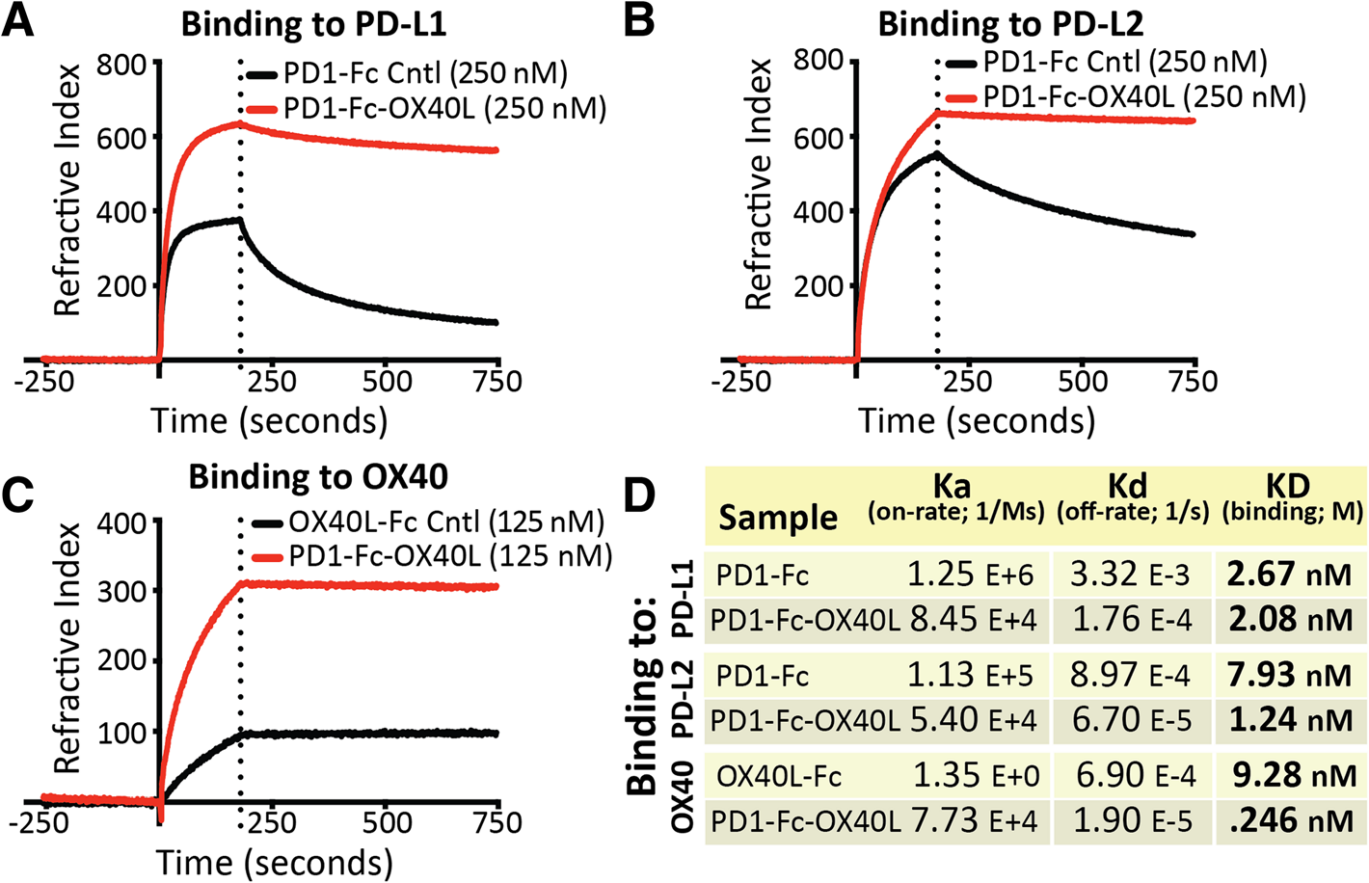
Human PD1-Fc-OX40L ARC binds with high affinity to PD-L1, PD-L2, and OX40. On-rate (Ka), off-rate (Kd) and binding affinity (KD) were determined by surface plasmon resonance for PD1-Fc-OX40L binding to chip immobilized (**a**) PD-L1-His, (**b**) PD-L2-His, and (**c**) OX40-His. Recombinant human PD1-Fc and OX40L-Fc were used as controls for binding. (**d**) Summary of binding results depicted in the graphs (**a-c**)

The binding affinity of PD1-Fc-OX40L was characterized to each cognate ligand and receptor by surface plasmon resonance (Fig. 2a-d and Additional file 3: Figure S2C-E). These studies demonstrate that the PD1 domain of the ARC fusion protein retained high affinity binding to both PD-L1 (2.08 nM) and PD-L2 (1.24 nM), similar to the recombinant PD1-Fc fusion protein control (2.67 nM and 7.93 nM, respectively) (Fig. 2a-b,d). The binding affinity of the OX40L domain of the ARC fusion protein to OX40 was measured to be considerably higher (0.246 nM) than a recombinant OX40L-Fc fusion protein control (9.28 nM) (Fig. 2c-d). The PD1-Fc-OX40L ARC was also observed to have a considerably longer off-rate than control proteins when the dissociation from PD-L1 (18 fold longer), PD-L2 (13.4 fold longer) and OX40 (36.32 fold longer) was determined (Fig. 2d). We speculate that this may be related to an avidity effect transmitted by the OX40L and Fc regions to the PD1 domain of the fusion protein, since PD1 normally exists and functions as a monomeric protein. Interestingly, PD1-Fc-OX40L was shown to bind the neonatal Fc receptor (FcRn), but not to FcγRI, FcγRIIb or FcγRIII (Additional file 3: Figure S2C-E and data not shown). These properties suggest that PD1-Fc-OX40L may have little to no Fc effector function, but instead, FcRn binding may beneficially contribute to increased half-life in vivo [21].

### Binding to native PD-L1 and OX40

**Fig. 3:**
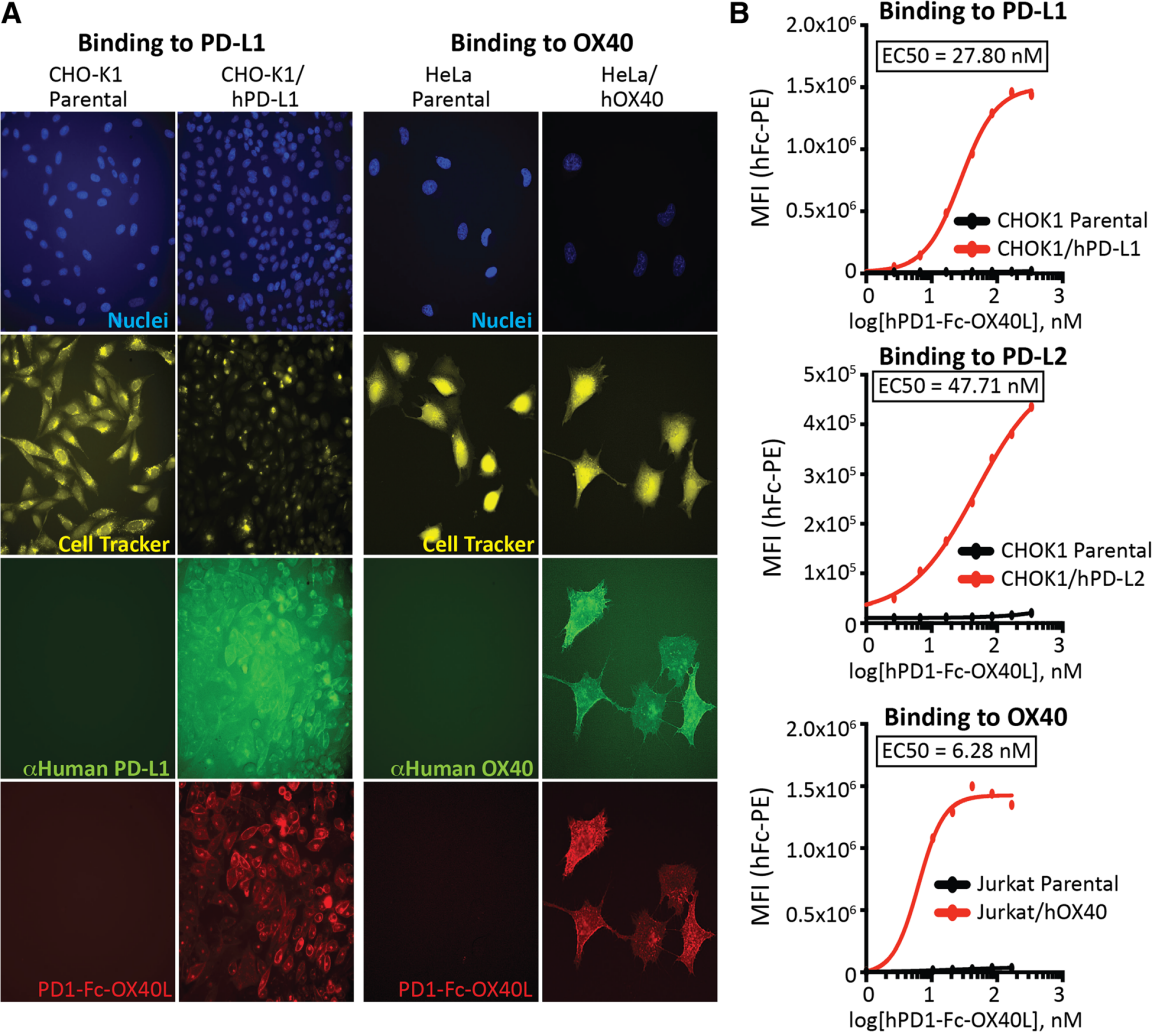
Human PD1-Fc-OX40L ARC binds efficiently to cells expressing PD-L1, PD-L2, and OX40 in vitro and ex vivo. The in vitro cell binding affinity of the human PD1-Fc-OX40L ARC was assessed using stable cell lines engineered to express high-levels of human PD-L1 (CHOK1/hPD-L1), human PD-L2 (CHOK1/hPD-L2), and human OX40 (Jurkat/hOX40 and HeLa/hOX40). **a** Representative immunofluorescence and (**b**) flow cytometry binding analysis to PD-L1, (top) PD-L2 and (middle) OX40 (bottom) expressing cells, generating dose-response plots to derive EC50 values

To further characterize PD1-Fc-OX40L, binding studies were performed to show specific interaction with PD-L1/L2 (CHOK1 parental, CHOK1/hPD-L1, and CHOK1/hPD-L2) and OX40 (Jurkat parental, Jurkat/hOX40, HeLa parental, and HeLa/hOX40) expressed in mammalian cell membranes, in cell lines developed to stably over-express the appropriate ligand or receptor (Additional file 4: Figure S3A-B). Immunofluorescence (IF) was used to visualize Alexa Fluor labeled PD1-Fc-OX40L and its co-localization with fluorescently labeled anti-PD-L1 or anti-OX40 (Fig. 3a). Interestingly, PD1-Fc-OX40L binding to PD-L1 appears to distribute uniformly across the entire cell surface, whereas OX40 binding appears as punctate clusters through the membrane. Both binding patterns are consistent with known affinity/avidity characteristics; namely monomeric binding of PD1 to PD-L1/L2 and multimeric binding of OX40L to OX40 receptor, indicative of the PD1-Fc-OX40L driving OX40 clustering, which is known to be critical for efficient signaling [22]. To determine the binding kinetics of the PD1 domain of PD1-Fc-OX40L, CHOK1/hPD-L1 and CHOK1/hPD-L2 cells were incubated in vitro with the PD1-Fc-OX40L ARC, generating an EC50 of 27.8 nM and 47.71 nM for PD-L1 and PD-L2, respectively (Fig. 3b and Additional file 4: Figure S3B). To determine binding of the OX40L domain of PD1-Fc-OX40L, Jurkat/hOX40 cells were incubated with the ARC. This interaction produced EC50 values of 6.28 nM. CHOK1 parental cells and Jurkat cells were used to demonstrate specificity of ARC binding to its intended targets. Primary human T cells were also stimulated with PMA/PHA/ionomycin to upregulate OX40 (Additional file 4: Figure S3C), and PD1-Fc-OX40L was shown to have increased binding to activated human CD4+ and CD8+ T cells as compared to non-activated human T cells (Additional file 4: Figure S3D). Binding of PD1-Fc-OX40L to human tumor cell lines differentially expressing PD-L1 and PD-L2 was also examined. While the ARC molecule bound specifically to the PD-L1_high_ HCC827, non-small cell lung cancer cell line in a concentration dependent manner, binding was not observed to the PD-L1_low_, PC3 prostate cancer cell line (Additional file 5: Figure S4A-B).

### Immunogenicity assessment of PD1-fc-OX40L

Human PD1-Fc-OX40L was constructed from native amino acid sequences, and was predicted to have a low probability of immunogenicity based on in silico modeling (iTope platform from Abzena, data not shown). To expand upon this analysis, we performed a comprehensive ex vivo assessment of CD4+ T cell response. PBMCs from 50 healthy human donors were depleted of CD8 T cells and incubated in serum-free media with .3 μM of the human PD1-Fc-OX40L ARC, the neoantigen positive control KLH, or a clinical stage molecule that serves as an example of a non-immunogenic molecule (Exenatide). PD1-Fc-OX40L did not cause proliferation nor stimulate IL-2 production (via ELISpot) of CD8-depleted PBMCs from 50 individual human donors above the background control, consistent with a lack of ex vivo immunogenicity (Additional file 4: Figure S3E-F).

### Human PD1-fc-OX40L functional activity

**Fig. 4:**
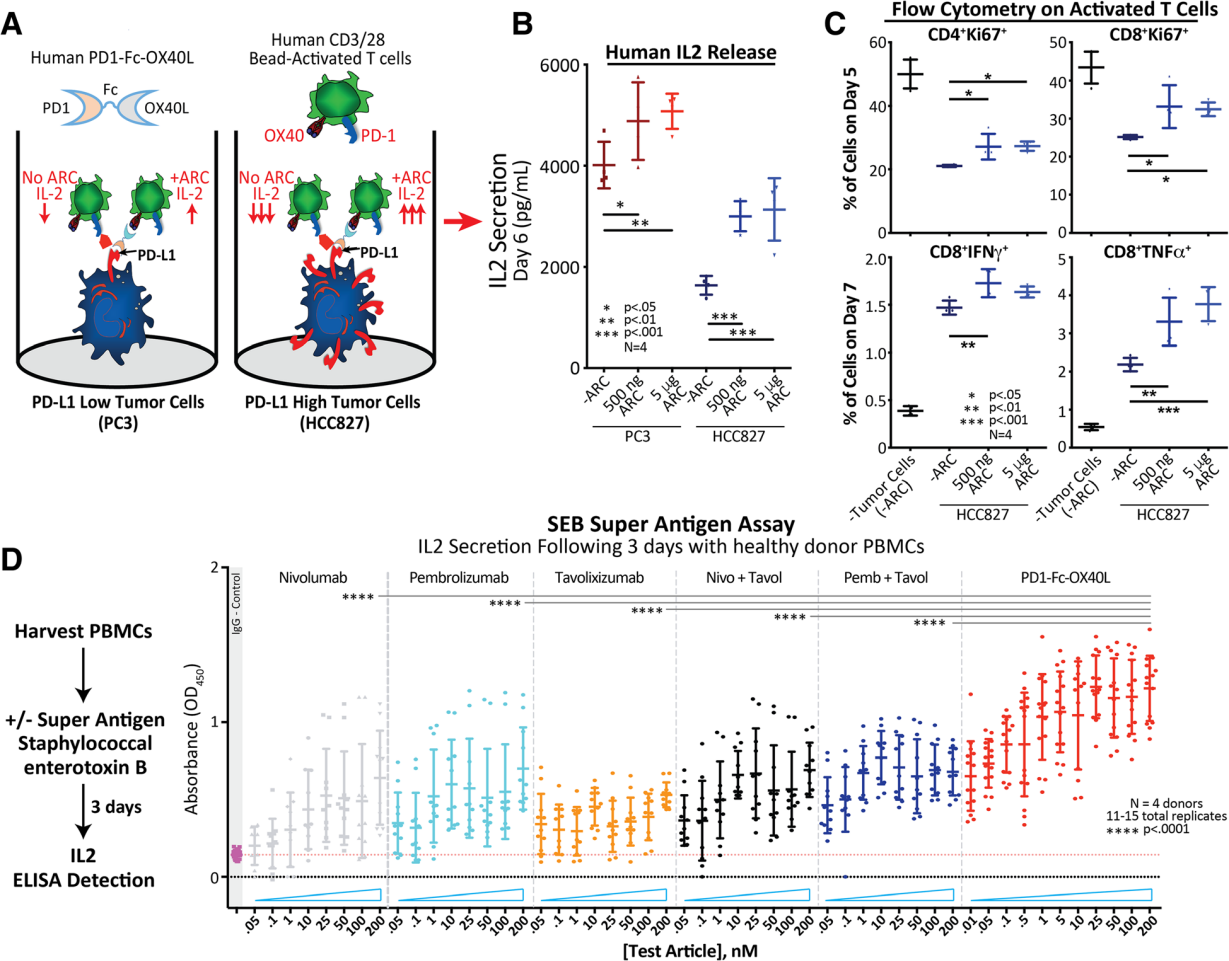
The human PD1-Fc-OX40L ARC has functional activity in vitro and ex vivo in both tumor co-culture and SEB super-antigen assays. **a** Schematic of the tumor/T cell co-culture assay. CD3+ human T cells stimulated for 48 h with suboptimal levels of CD3/CD28/IL-2, were plated on mitomycin-c treated PD-L1_low_ (PC3) and PD-L1_high_ (HCC827) tumor cells ± the PD1-Fc-OX40L ARC, for an additional 3–5 days (days 5–7 of the entire time-course). **b** On day 6 of the assay, culture supernatant was collected and analyzed by human IL-2 ELISA. **c** On days 5 (top) and 7 (bottom) of the assay, floating T cells were collected and subjected to extra- and intra-cellular flow cytometry in order to assess proliferation (Ki67) and markers of T cell activation (IFNγ & TNFα). **d** Workflow of the SEB super-antigen assay. Total primary human PBMCs were harvested and treated with *Staphylococcal* enterotoxin B ± the PD1-Fc-OX40L ARC and benchmark antibody controls. Culture supernatants were collected 3 days later and assessed for secreted levels of IL-2 by ELISA

To examine the functional activity of hPD1-Fc-OX40L, three separate in vitro functional assays were developed using primary human T cells or OX40 expressing NFkB-luciferase reporter cell lines. First, CD3+ T cells were isolated from human peripheral blood leukocytes and then sub-optimally stimulated with CD3/CD28 T cell activator beads (to upregulate PD1 and OX40), before being co-cultured with either PC3 cells (PD-L1_low_) or HCC827 cells (PD-L1_high_), in the presence or absence of PD1-Fc-OX40L to assess IL2 release (Fig. 4a). These studies demonstrated that IL-2 secretion from CD3+ T cells was reduced in the presence of tumor cells expressing high levels of PD-L1, but could be restored through addition of PD1-Fc-OX40L (Fig. 4b). Further analysis of the human T cells from this assay by intracellular flow cytometry also demonstrated that PD1-Fc-OX40L stimulated increased expression of Ki67, an intracellular marker for cell proliferation, in both CD4+ and CD8+ T cells, and increased expression of IFNγ and TNFα in human CD8+ T cells (Fig. 4c). While PD-L2 expression was detected on both tumor cell lines, albeit with a relatively higher level on HCC827 cells, the effect of PD1-Fc-OX40L on IL-2 secretion appears to correlate with the level of PD-L1 expression on the tumor cells (Additional file 5: Figure S4A).

In a second functional assay, to determine the relative potency of PD1-Fc-OX40L to sequence equivalents of commercial human antibody therapeutics, human leukocytes were incubated with increasing concentrations of the superantigen, *Staphylococcus* enterotoxin B (SEB) in the presence of pembrolizumab (pembro; αPD1), nivolumab (nivo; αPD1), tavolixizumab (tavol; αOX40), the combination of pembro/tavol, the combination of nivo/tavol – equivalents -, or PD1-Fc-OX40L (Fig. 4d). PD1-Fc-OX40L stimulated higher levels of IL-2 secretion in the presence of SEB compared with any of the antibody controls that were incubated individually or in combination (Fig. 4d). Increased IL-2 secretion was determined to be on a per-cell basis, as PBMCs did not proliferate significantly during the course of the 3 day experiment (Additional file 5: Figure S4D-E). Additionally, the SEB assay was then performed to compare PD1-Fc-OX40L with commercially available single-sided fusions, including PD1-Fc, Fc-OX40L, and the combination of the two (Additional file 5: Figure S4F). PD1-Fc-OX40L demonstrated increased IL-2 secretion compared to the single-sided fusions or a combination of the two, which was determined to be primarily dependent on CD4+ T cells (Additional file 5: Figure S4F-G). These data suggested that either the physical tethering of both checkpoint-blocking and immune-stimulating signals provided a mechanistic advantage greater than either signal given separately, or that the oligomeric nature of OX40L in the PD1-Fc-OX40L construct provided an avidity advantage distinct from the comparator antibodies. To determine the contribution of the individual ARC domains (PD1 and/or OX40L) to overall SEB stimulating IL-2 activity, a K78A mutation was introduced into the mouse PD1-Fc-OX40L sequence to generate a mPD1(K78A)-Fc-OX40L mutant protein that lacked the capacity to bind PD-L1 and PD-L2 [18, 19]. The mPD1(K78A)-Fc-OX40L ARC was indistinguishable from the wild-type (WT) ARC in Fc and OX40L specific ELISAs, but was unable to bind PD-L1, PD-L2, or function in the dual binding ELISA (Additional file 1: Figure S5K). When compared head to head with the WT ARC, the K78A mutant demonstrated equivalent IL-2 secretion in the SEB assay(Additional file 1: Figure S5L), suggesting that enhanced function of the OX40L domain of PD1-Fc-OX40L, and not purely colocalization/membrane tethering via PD1/PD-L1 interaction, were responsible for improved function.

To provide further evidence that PD1-Fc-OX40L stimulates a signaling cascade downstream of OX40, an OX40-dependent NFkB-luciferase reporter assay was set up using Jurkat cells (Additional file 5: Figure S4C). Because OX40 signaling is dependent upon TCR activation, CD3 stimulation (with or without concurrent CD28 stimulation) is required to stimulate basal activation of NFkB-luciferase. Addition of a control Fc-OX40L fusion protein was then shown to further stimulate NFkB-luciferase activity in a concentration dependent manner (Additional file 5: Figure S4C). Consistently, addition of PD1-Fc-OX40L was shown to stimulate the NFkB-luciferase reporter in a concentration dependent manner. It is important to note that Fc receptors were absent in this assay, indicating that Fc receptor cross-linking was not required for functional activity from PD1-Fc-OX40L.

### Murine PD1-fc-OX40L characterization and anti-tumor activity

The murine equivalent of PD1-Fc-OX40L, hereafter referred to as mPD1-Fc-OX40L, was produced and characterized similar to the human ARC protein and used for in vivo analysis due to the fact that the human ARC was not cross-reactive with murine PD-L1 and OX40 (data not shown). mPD1-Fc-OX40L demonstrated high affinity binding to mPD-L1 and mOX40 targets across in vitro binding assays and displayed biological activity as evidenced by increased IL2 release in tumor/T cell co-culture and SEB assays (Additional file 1: Figure S5A-J). The CT26 mouse colon tumor model is known to respond to antibody mediated PD1 blockade, and was therefore utilized to assess the anti-tumor efficacy of mPD1-Fc-OX40L [23].

**Fig. 5:**
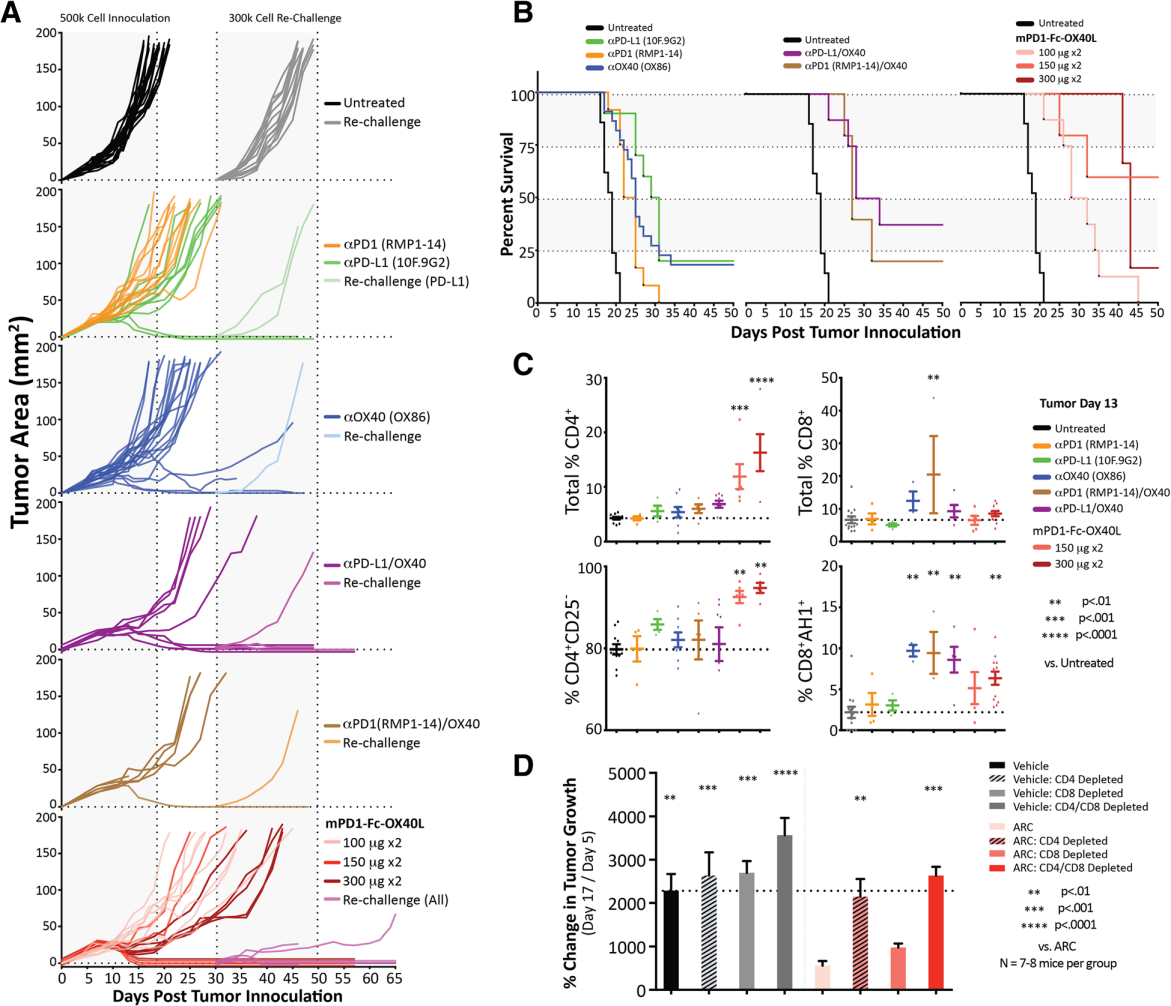
Mouse PD1-Fc-OX40L ARC is efficacious at treating CT26 colon cancer tumors and increasing survival. **a** Mice were inoculated subcutaneously on the rear flank with 5 × 10^5^ CT26 cells on day 0, and then treated with 2 doses (all by IP injection on days 5 & 7 once the tumors established and were ~ 4–6 mm in diameter), consisting of 100 μg for each antibody dose and either 100 μg, 150 μg or 300 μg for each dose of the murine PD1-Fc-OX40L ARC. Each line represents the tumor growth from an individual mouse. The first dotted line (~day 18) represents the mean when all untreated mice reached tumor burden. On day 30, surviving mice were challenged with a secondary tumor consisting of 3 × 10^5^ cells on the opposing rear flank. Thirteen new untreated mice were also inoculated on day 30 as a control for tumor re-challenge growth. All ARC treated mice that survived until the day 30 challenge, were combined when plotting the ‘re-challenge’ tumor growth curves. **b** Kaplan-Meier curves were generated for each treatment group, and are plotted identically in order to directly compare sample groups. **c** A cohort of mice was euthanized 13 days following tumor inoculation, and tumors were excised, dissociated, and subjected to immune phenotyping by flow cytometry. Total populations of CD4^+^ and CD8^+^ T cells were assessed, as well as CD4^+^CD25^−^ effector and CD8^+^AH1^+^ antigen-specific cytotoxic cells. **d** Some mice were treated on days − 1, 1 and 10 with CD4, CD8, or a combination of the two, depleting antibodies. CT26 tumors were again inoculated on day 0, and mice were treated with two IP treatments of 300 μg of the mPD1-Fc-OX40L ARC on days 5 and 7. The percent change in tumor growth between the first treatment day (day 5) and day 17 is shown

CT26 colon carcinoma cells were implanted in the hind flank of Balb.c mice and tumors were allowed to become established before treatment was initiated with mPD1-Fc-OX40L or with the indicated benchmark antibodies given alone or in combination (Fig. 5). Three increasing doses of mPD1-Fc-OX40L were compared, and the higher doses (150 μg × 2 and 300 μg × 2) were shown to be protective and outperform the lower dose group (100 μg × 2). Even in the low-dose group, however, tumor control was significantly better than monotherapy antibody treatment to PD1 (RMP1–14), PD-L1 (10F.9G2) or OX40 (OX86) and equivalent to the antibody combinations (Fig. 5a-b). Increased survival and overall tumor rejection was observed when mPD1-Fc-OX40L was administered at either 150 μg or 300 μg per injection (Fig. 5b, Additional file 6: Figure S6A-B). Serum cytokine analysis was performed on CT26 bearing mice treated with antibodies and the mPD1-Fc-OX40L ARC (Additional file 6: Figure S6c-d). The serum cytokine response on day 13 was variable between groups, but seemed to be partially predictive of response, and with respect to mPD1-Fc-OX40L treatment, serum IFNγ concentrations increased in a dose dependent manner (Additional file 6: Figure S6D). A cohort of treated mice was euthanized 13 days after initial tumor inoculation. Tumors and spleens were isolated, dissociated, and analyzed for proportions of effector, non-effector/Treg, and antigen-specific CD8+ (AH1-tetramer) populations by flow cytometry (Fig. 5c and Additional file 6: Figure S6F). Treatment with the mPD1-Fc-OX40L ARC led to an increase in CD4+ T cell populations in both the tumor (Fig. 5c) and spleen (Additional file 6: Figure S6F), that were enriched for effector T cells (CD4^+^CD25^−^) compared with non-effector/Treg (CD4^+^CD25^+^ or CD4^+^FOXP3^+^) cells. In fact, the ratio of CD4+ effector to Treg cells in mPD1-Fc-OX40L treated mice was 2–3-fold higher than antibody combinations in the tumor and ~ 1.5-fold higher in the spleen. Additionally, PD1-Fc-OX40L led to a significant increase in antigen-specific CD8 T cells within the tumor. The higher doses of mPD1-Fc-OX40L, which remain below the total dose of antibody administered, improved the rate of complete primary tumor rejection and were nearly 2–3 fold higher than was observed with two distinct combinations of PD1/L1 and OX40 antibodies. Interestingly, the rate of rejection of secondary tumors was approximately 75% with mPD1-Fc-OX40L in the absence of any re-treatment compared to the controls (Additional file 6: Figure S6A; summary of CT26 tumor results).

The anti-tumor activity of OX40 has been reported to be primarily dependent upon CD4+ T cells [24, 25]. In vitro studies suggested an important role for CD4+ T cells for PD1-Fc-OX40L. To further explore the requirement of CD4 and/or CD8 T cells for overall mPD1-Fc-OX40L anti-tumor efficacy tumor studies were conducted in mice wherein CD4+ cells, CD8+ cells, or both populations were depleted using monoclonal antibodies.. Mice depleted of either CD4+ T cells or the combination of CD4+/CD8+ T cells, were unable to mount an effective anti-tumor response (Fig. 5d and Additional file 6: Figure S6E). These findings were consistent with the CD4-dependent activity of human PD1-Fc-OX40L in the SEB assay (Additional file 5: Figure S4G). In contrast, ARC activity in mice treated with CD8 depleting antibodies was only mildly reduced.

### PD1-fc-OX40L stimulates tumor cell killing in vitro

**Fig. 6:**
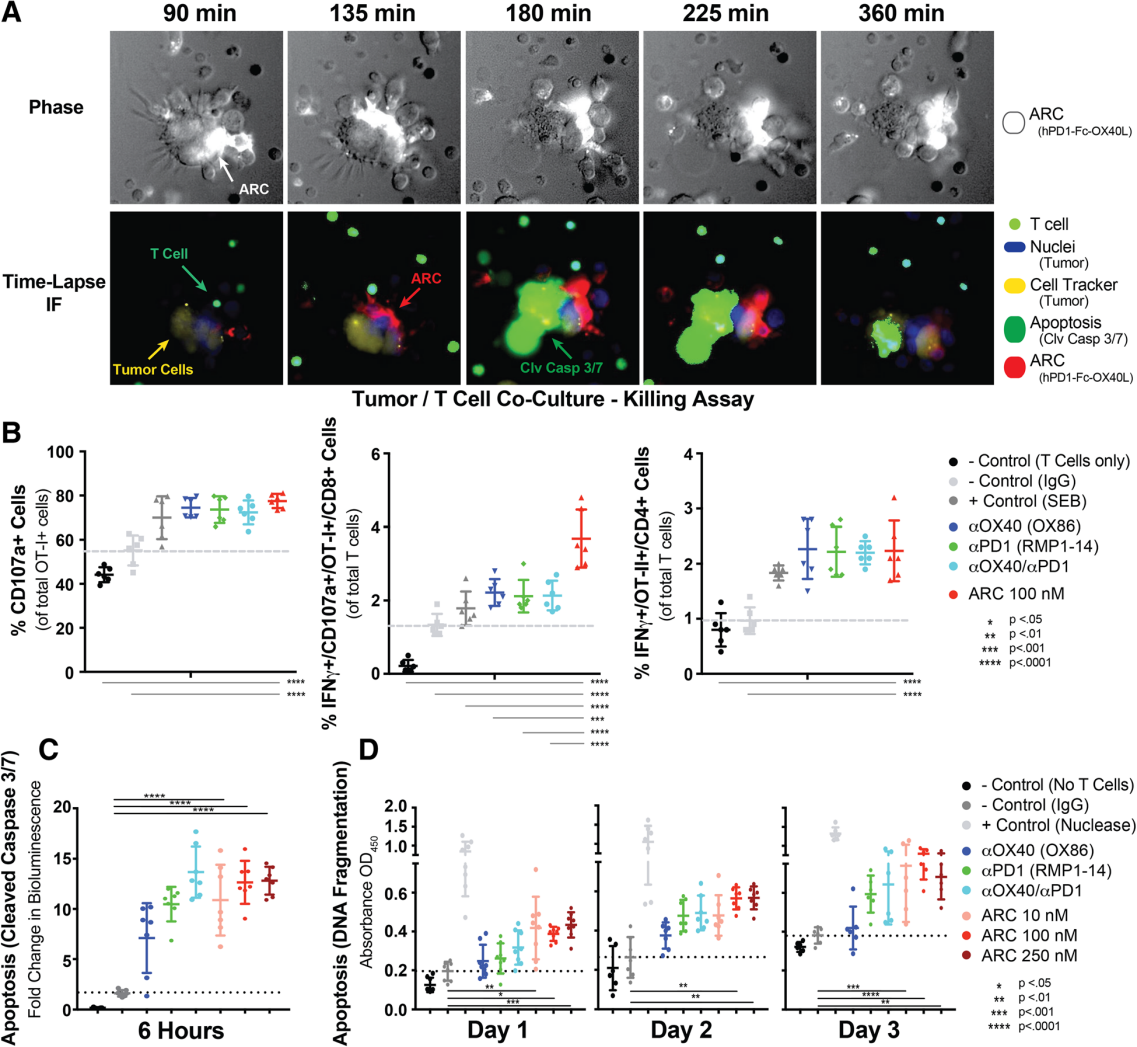
PD1-Fc-OX40L Stimulates Tumor Cell Killing In Vitro. **a** Human NCI-H2023 cells were co-cultured with human T cells stimulated for 2 days with sub-optimal CD3/CD28/IL2. Tumor cells were labeled with a cell tracker (yellow) and nuclear stain (blue), and then cultured with 150 nM of the human ARC, labeled with AF647 (Red). A cleaved caspase-3/7 reagent (green) was added to cells and then the same field was imaged over a time-course. T cells also fluoresced in the green channel and can be differentiated from tumor cells in the phase images (top images) by morphology. Similar to the red channel, the phase images also read out ARC staining (white). Mouse T cells were isolated from C57BL/6 mice (adoptively transferred with OT-I/OT-II cells and vaccinated with Ova/Alum) and co-cultured with B16.F10-ova melanoma cells in the presence or absence of OX40 agonist antibody (OX86), PD-1 blocking antibody (RMP1–14), the combination of those two antibodies or the PD1-Fc-OX40L ARC. **b** Following 24 h of culture, T cell degranulation was measured by analyzing the proportion of OT-I+/CD8+ T cells expressing CD107a on the cell surface, and IFNγ intracellularly; in both OT-I+/CD107a + cells and also in OT-II+ cells. **c** Induction of caspase 3/7 cleavage was also evaluated in B16.F10-ova cells in each condition following 6 h of co-culture. **d** As a late-stage indicator of tumor cell death, a TUNEL assay was performed on days 1, 2 and 3 of co-culture. Results indicate mean ± SD for ≥2 replicates per condition for each of 2 separate experiments

To investigate whether PD1-Fc-OX40L improves anti-tumor immunity through direct or indirect effects on T cell mediated killing, a series of in vitro tumor cell killing assays were performed using human PD1-Fc-OX40L. First, in order to confirm bi-functional mechanism of action (Additional file 2: Figure S1D-F); more specifically – the ability of PD1-Fc-OX40L to physically ‘tether’ tumor cells with T cells and stimulate potent cytotoxic activity, we used the visual tool of time-lapse immunofluorescent live cell imaging (Fig. 6a). The PD-L1 expressing human non-small cell lung cancer cell line, NCI-H2023 was co-cultured with human CD3+ T cells that were stimulated for 2 days with suboptimal concentrations of CD3, CD28, and IL2. Prior to co-culture, the tumor cells were labeled with a cell tracker and nuclear stain, and pre-treated with 150 nM of the human PD1-Fc-OX40L ARC that was fluorescently labeled with an Alexa Fluor 647 fluorochrome. Interestingly, as T cells migrated into close proximity of tumor cells, the density of detectable ARC (Fig. 6a; visualized as white in the phase and red in the IF) increased significantly, suggesting that the ARC is able to coat PD-L1 on the tumor cell surface, and then functionally cluster OX40 receptors on the T cell at the physical interface between an interacting T cell and tumor cell. T cells can be visualized in Fig. 6a based on their small size, distinct morphology, and the fact that many of them autofluoresced. Tumor cells are visualized by their larger size and the presence of blue nuclear stain and yellow cell tracker dye. Remarkably, as one T cell (green arrow) migrates close to a group of tumor cells, ARC signal between the two cells increases in intensity (Fig. 6a; red arrow, progressing from 90 to 180 min). This increase in ARC localization was soon followed by a burst of apoptotic activity in the tumor cells (green arrow), from 180 to 360 min. This phenomenon was observed across multiple fields/wells, and provided direct evidence that the role of the PD1-Fc-OX40L ARC was indeed bi-functional and exerted potent tumor-killing activity through simultaneous interaction with T cells.

To quantitate this tumor killing activity, we deployed three assays commonly used to assess cell death/killing. The first of these assays was a CD107a T cell degranulation assay, in which lysosomal-associated membrane glycoproteins (i.e. CD107a or LAMP-1), migrated to the cell surface at the T cell/tumor cell synapse and facilitated cytolytic activity [26]. The second assay quantified the cleavage of the effector caspases 3 and 7, involved in apoptotic cell death (as was visualized in Fig. 6a above) [27]. Finally, a high-throughput terminal deoxylnucleotidyl transferase dUTP (TUNEL) assay was used to quantitate endonuclease driven DNA fragmentation during cell death [28]. These are each complimentary methods, wherein the CD107a degranulation assay informs on the potential killing capacity of an activated T cell, the caspase 3/7 cleavage assay provides an early readout as to whether cell death pathways have been activated in a target cell, and the TUNEL assay informs on whether activation of those cell death pathways was followed by damage to genomic DNA (Fig. 6b-d and Additional file 6: Figure S6H-J).

Primary mouse T cells, isolated from mice that had been adoptively transferred with OT-I (CD8+) and OT-II (CD4+) cells and vaccinated with Ova/alum, were co-cultured with B16.F10 tumor cells expressing ovalbumin (B16.F10-ova). In each co-culture, the activity of OX40 agonist antibodies, PD-1 blocking antibodies and the combination of those two antibodies was compared to mPD1-Fc-OX40L. These results demonstrated that both OX40 agonist and PD-1 blocking antibodies led to increased CD107a degranulation in CD8+ T cells (Fig. 6b) compared to the IgG control, which was followed by cleavage of caspases 3 and 7 in the B16.F10-ova tumor cells (Fig. 6c) and DNA fragmentation (Fig. 6d) as compared to both the ‘no T cell’ and unstimulated controls. Across each assay, there was increased evidence of T cell mediated tumor killing when the two antibodies were added in combination, however those values did not reach significance over either antibody used as monotherapy. Treatment with mPD1-Fc-OX40L led to a similarly significant increase in CD107a degranulation and increased antigen-specific/IFNγ+ CD4 T cells (Fig. 6b, right graph), caspase 3/7 cleavage, and DNA fragmentation over negative controls (Fig. 6b-d). Interestingly, although the mPD1-Fc-OX40L ARC produced a similar increase in CD107a+/OT-I/CD8 cells as compared to the monotherapy or combined antibodies, there were significantly more IFNγ+ cells within this population following ARC treatment (Fig. 6b, middle graph). These data indicate that PD1-Fc-OX40L directly increases the cytotoxic potential of murine CD4+/CD8+ T cells and subsequent killing of murine tumor cells.

## Discussion

Since 1998, Enbrel (TNFRSF1b-Fc fusion protein) has been a marketed product for the treatment of autoimmune disease. Similarly, fusion proteins incorporating TNF superfamily ligands were reported in the scientific literature since 1991 [29]. Based on the functionality of these two classes of proteins, we endeavored to construct a single fusion protein that could bridge ECDs from distinct type I and type II membrane proteins. In this report we have described the generation and characterization of the first ARC construct, PD1-Fc-OX40L, for both mouse and human. It was unclear how such a construct would fold, whether it could be secreted from cells, purified by affinity chromatography or retain functional binding to each cognate receptor and ligand. The data presented show that PD1-Fc-OX40L can be produced, purified, and shown to bind PD-L1/L2 and OX40 simultaneously and with high affinity. PD1-Fc-OX40L led to functional activation of mouse and human T cells in vitro, and significantly outperformed PD-1/L1 blockade as monotherapy, OX40 agonist monotherapy and even, surprisingly, the combination of antibody mediated PD-1/L1 blockade combined with OX40 agonist therapy [30, 31].

Despite the fact that both PD1-Fc-OX40L and PD-1(L1) blocking and OX40 agonist antibodies target the same biological pathways, there are potential differences in the mechanism of action when engagement of those pathways is targeted to the tumor microenvironment. When two separate antibodies targeting PD-1(L1) and OX40 are administered by i.p. or i.v. infusion, the two antibodies are free to distribute independently from one another immediately following infusion.

The primary site of activity for PD-1(L1) blocking antibodies is within the tumor microenvironment. OX40 may act in many tissues, however this receptor and pathway are optimally activated in T cells in the setting of coordinated antigen and costimulatory molecule expression [17, 32, 33]. In the case of tumor immunity, this suggests that specific activation of OX40 on the surface of T cells that are actively engaging with a tumor cell may lead to superior activity than engagement of OX40 on the surface of T cells that are in circulation or that are residing in other tissues. Further, because PD-1 engagement leads to direct inhibition of T cell receptor signaling [15, 34], coordinated blockade or PD-1(L1) at the time of T cell activation is more likely to synergize with OX40 activation than when those two signals are uncoupled from one another. In addition, the agonist activity of antibodies targeting TNF superfamily receptors (including OX40) frequently requires Fc receptor mediated cross-linking [12, 35–37]. When multiple separate antibodies are administered, each with the potential to bind Fc receptors, competition for Fc receptor binding may directly reduce the agonist potential of the TNFR agonist antibody being administered; this possibility was unfortunately not controlled for in two recent studies investigating the sequencing of PD1 and OX40 antibodies (both of which were rat IgGs) [30, 31].

In the case of PD1-Fc-OX40L, the OX40L signal is physically tethered to the PD-1 domain via the Fc region, but provides receptor agonism that is fully independent from Fc receptor cross-linking. This difference between PD1-Fc-OX40L and OX40 agonist antibodies may have an important contribution to therapeutic activity because competition for Fc receptor binding does not have to be considered in the dosing regimen for PD1-Fc-OX40L. Proper multimerization of TNFR agonist antibodies is critical to function in pre-clinical models, and inappropriate clustering via Fc receptor binding or other means may be a liability for TNFR agonist antibodies in the clinic [37–39]. The inherent oligomeric properties of PD1-Fc-OX40L were shown herein to have an important role in activating OX40 using a PD1 mutant (K78A), which lacked the ability to bind PD-L1 as well as using in vitro signaling assays where FcR expressing cells were not provided. Finally, the observation that the off-rate of this molecule from PD-L1 and PD-L2 is longer than is expected from typical antibodies or fusion proteins, may partially explain the benefit conferred by PD1-Fc-OX40L in terms of tumor rejection, as compared to PD-1(L1) and OX40 agonist antibodies.

The functional activity of PD1-Fc-OX40L was shown to be superior to PD1 blocking, OX40 agonist, or the combination of those antibodies in both murine tumor models and in vitro functional assays. Across these studies, there is evidence to support the conclusion that the oligomeric nature of PD1-Fc-OX40L led to more effective OX40 activation in T cells, and that the PD1 domain could provide tethering of the compound to PD-L1 expressing tumor cells. Which of these mechanisms is more important for tumor rejection remains unclear, however the predominant dependence of murine anti-tumor activity and human IL-2 production (SEB assay) on CD4+ cells is intriguing. Further elucidation of the time course of CD4+ versus CD8+ T cell activation, as well as the maturation profile of these activated cells during the development of immune memory, will be further studied.

In the human genome there are > 1400 type I and > 450 type II single-pass membrane protein encoding genes. All immune checkpoint proteins and cytokine receptors currently being investigated for cancer immunotherapy are type I membrane proteins and all TNF superfamily ligands are type II membrane proteins. Thus, within the field of oncology there are hundreds of type I/type II combinations that could be explored. Type I and type II membrane proteins modulate pathology in a broad range of human disease however, which extends far beyond oncology. Given that these chimeric fusion proteins are based upon naturally occurring human DNA sequences, there is no requirement for a discovery screen, humanization or affinity maturation to develop new ARC molecules. Further, because the proteins are encoded as a continuous DNA sequence, there is no need for post-production conjugation or coupling. Together, these advantages reduce the development time for new ARC fusion proteins substantially, which may lead to faster development of combination therapeutics in oncology as well as other disease categories.

## Conclusions

PD1-Fc-OX40L was developed to solve a key challenge in cancer immunotherapy, namely the development of single therapeutics that can simultaneously block immune checkpoint pathways and activate TNF receptors. Due to the requirement of TNF receptors to trimerize and/or hexamerize for effective signaling, a dual-sided Fc fusion protein was developed as an alternative to antibody-based scaffolds in order to produce a compound which could oligomerize without the need for exogenous cross-linking via Fc receptors. The data indicate that PD1-Fc-OX40L is able to bind PD-L1 and OX40 simultaneously, stimulate OX40 signaling in the absence of cross-linking, and provide improved control of tumor immunity as compared to combinations of PD1 and OX40 targeted antibodies.

## Additional files

Additional file 1: **Figure S5.** In depth characterization of the mouse PD1-Fc-OX40L ARC. (TIF 4630 kb)

Additional file 2: **Figure S1.** Agonist Redirected Checkpoint (ARC) fusion proteins link type I and type II membrane protein extracellular domains via a CH2-CH3, Fc region. (TIF 981 kb)

Additional file 3: **Figure S2.** Human PD1-Fc-OX40L ARC binds via Fc and OX40L domains using ELISA, and to the neonatal receptor FcRn using SPR. (TIF 1003 kb)

Additional file 4: **Figure S3.** Human in vitro cell line characterization and immunogenicity analysis. (TIF 1673 kb)

Additional file 5: **Figure S4.** Human tumor cell line characterization for PD-L1/L2 expression and PD1-Fc-OX40L ARC binding, SEB assay, and NFkB-luciferase reporter assay. (TIF 2037 kb)

Additional file 6: **Figure S6.** Mouse PD1-Fc-OX40L efficacy ± CD4/CD8 depletion in CT26 tumor model and schematics of tumor killing/apoptosis assays performed in Fig. 6. (TIF 2696 kb)

## Abbreviations

ANOVA: Analysis of Variance
ARC: Agonist Redirected Checkpoint
CHO: Chinese Hamster Ovary
ECD: Extracellular Domain
ELISA: Enzyme Linked Immunosorbent Assay
GFP: Green Fluorescent Protein
HRP: Horseradish Peroxidase
IF: Immunofluorescence
IMDM: Iscove’s Modified Dulbecco’s Medium
nM: Nanomolar
PBS: Phosphate Buffered Saline
pM: Picomolar
PMA: Phorbol 12-Myristate 13-Acetate
RT: Room Temperature
SEB: *Staphylococcus* enterotoxin B
TCR: T Cell Receptor
TNF: Tumor Necrosis Factor
TNFR: Tumor Necrosis Factor Receptor
TNFRSF: Tumor Necrosis Factor Receptor Superfamily
TUNEL: Terminal Deoxylnucleotidyl Transferase dUTP
