# Supplementary figures and images for: Agonist redirected checkpoint, PD1-Fc-OX40L, for cancer immunotherapy
Source: J Immunother Cancer. 2018 Dec 18;6:149. doi: 10.1186/s40425-018-0454-3 (PMC6299665; doi:10.1186/s40425-018-0454-3)

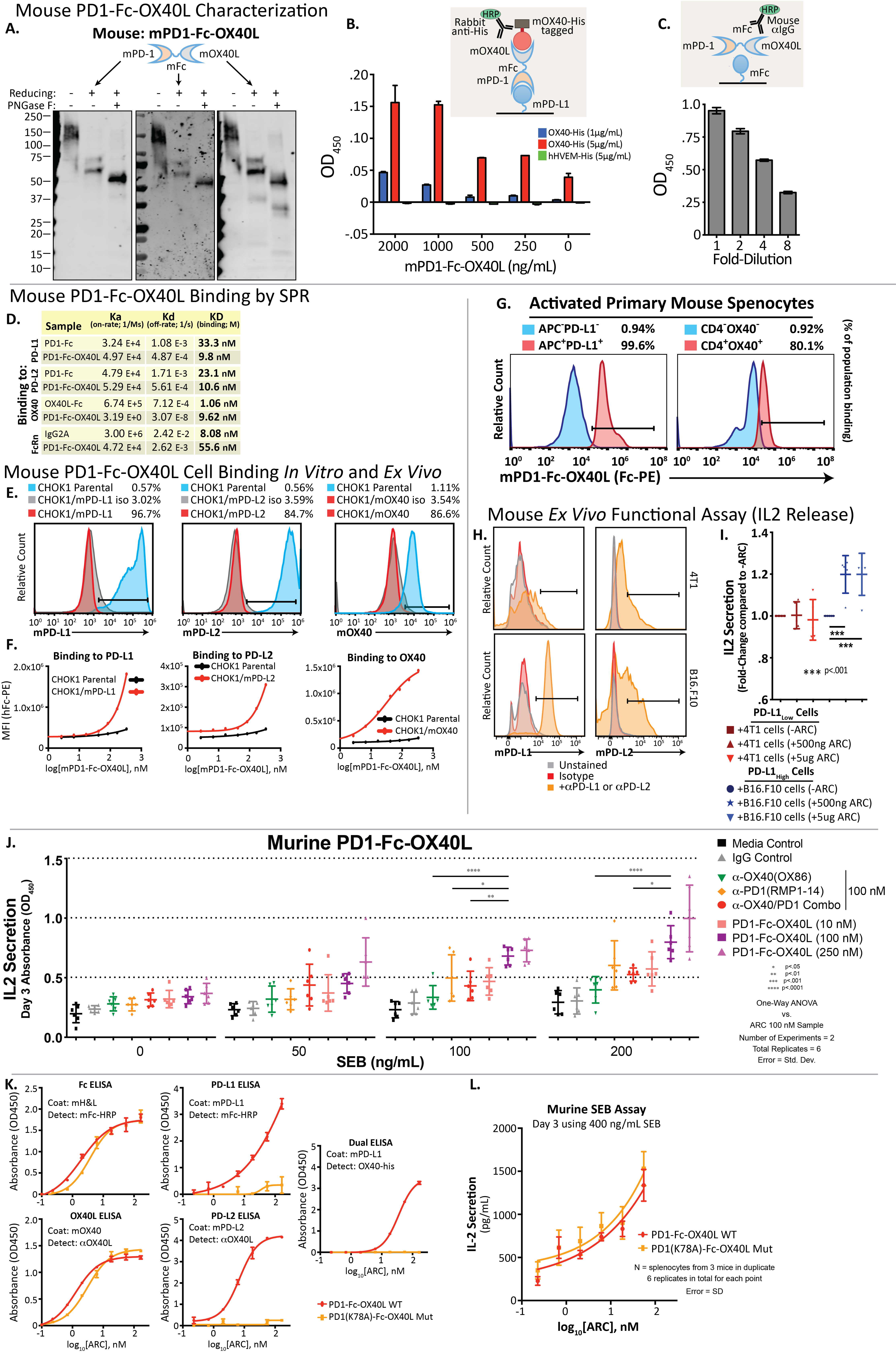

Supplement: Supplementary file 1 — Figure S5. In depth characterization of the mouse PD1-Fc-OX40L ARC. (TIF 4630 kb) [file 40425_2018_454_MOESM1_ESM.tif]

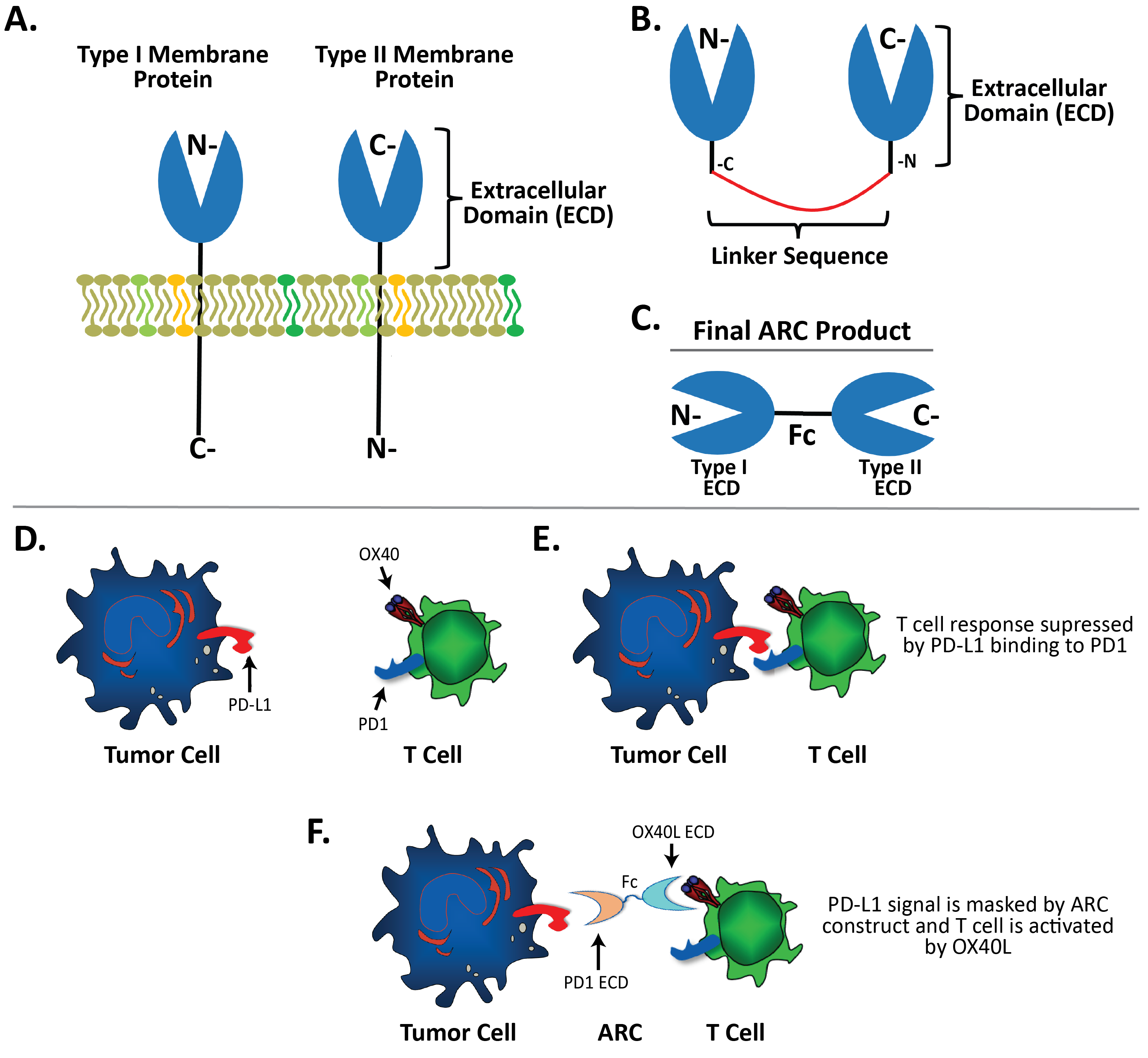

Supplement: Supplementary file 2 — Figure S1. Agonist Redirected Checkpoint (ARC) fusion proteins link type I and type II membrane protein extracellular domains via a CH2-CH3, Fc region. (TIF 981 kb) [file 40425_2018_454_MOESM2_ESM.tif]

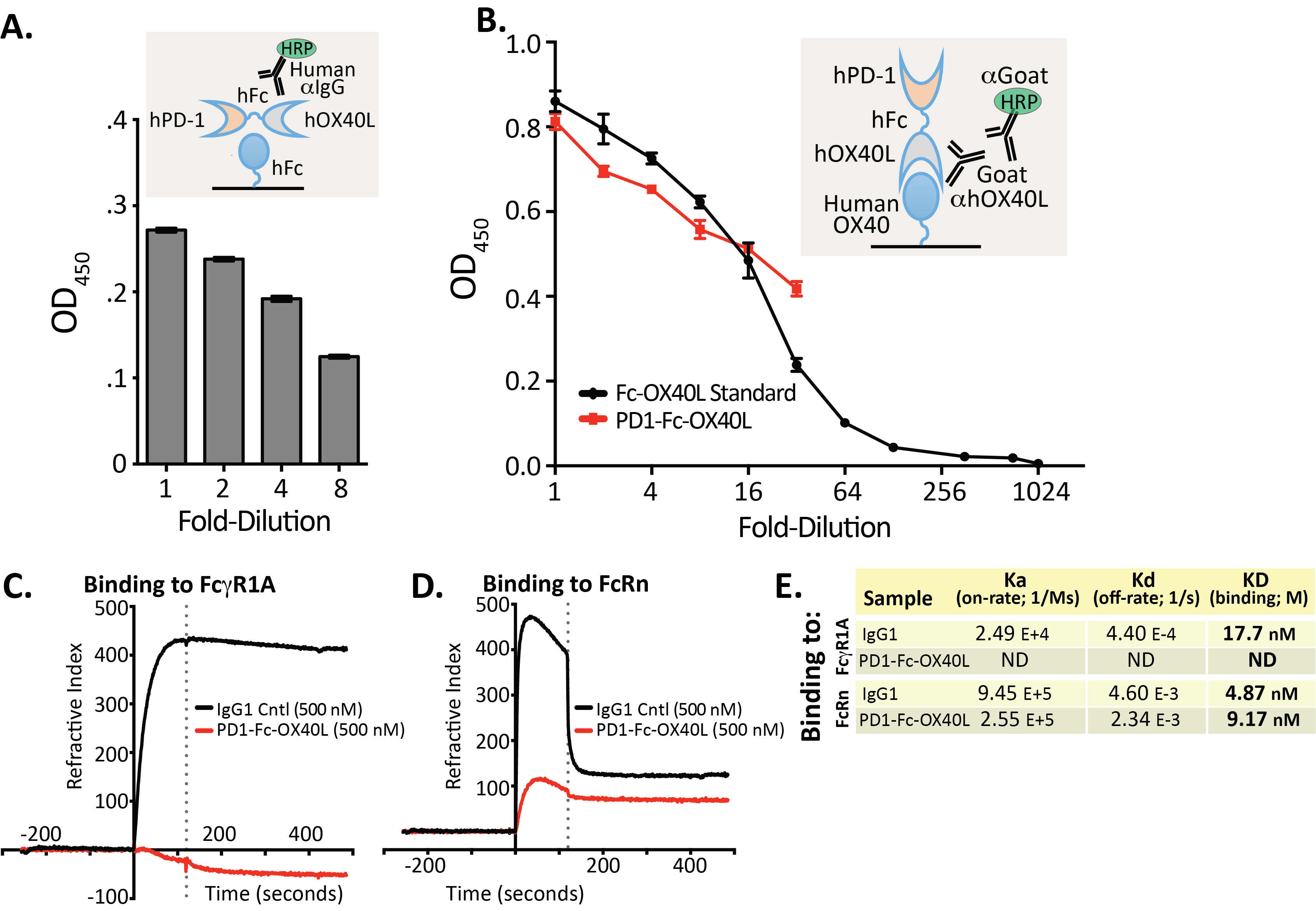

Supplement: Supplementary file 3 — Figure S2. Human PD1-Fc-OX40L ARC binds via Fc and OX40L domains using ELISA, and to the neonatal receptor FcRn using SPR. (TIF 1003 kb) [file 40425_2018_454_MOESM3_ESM.tif]

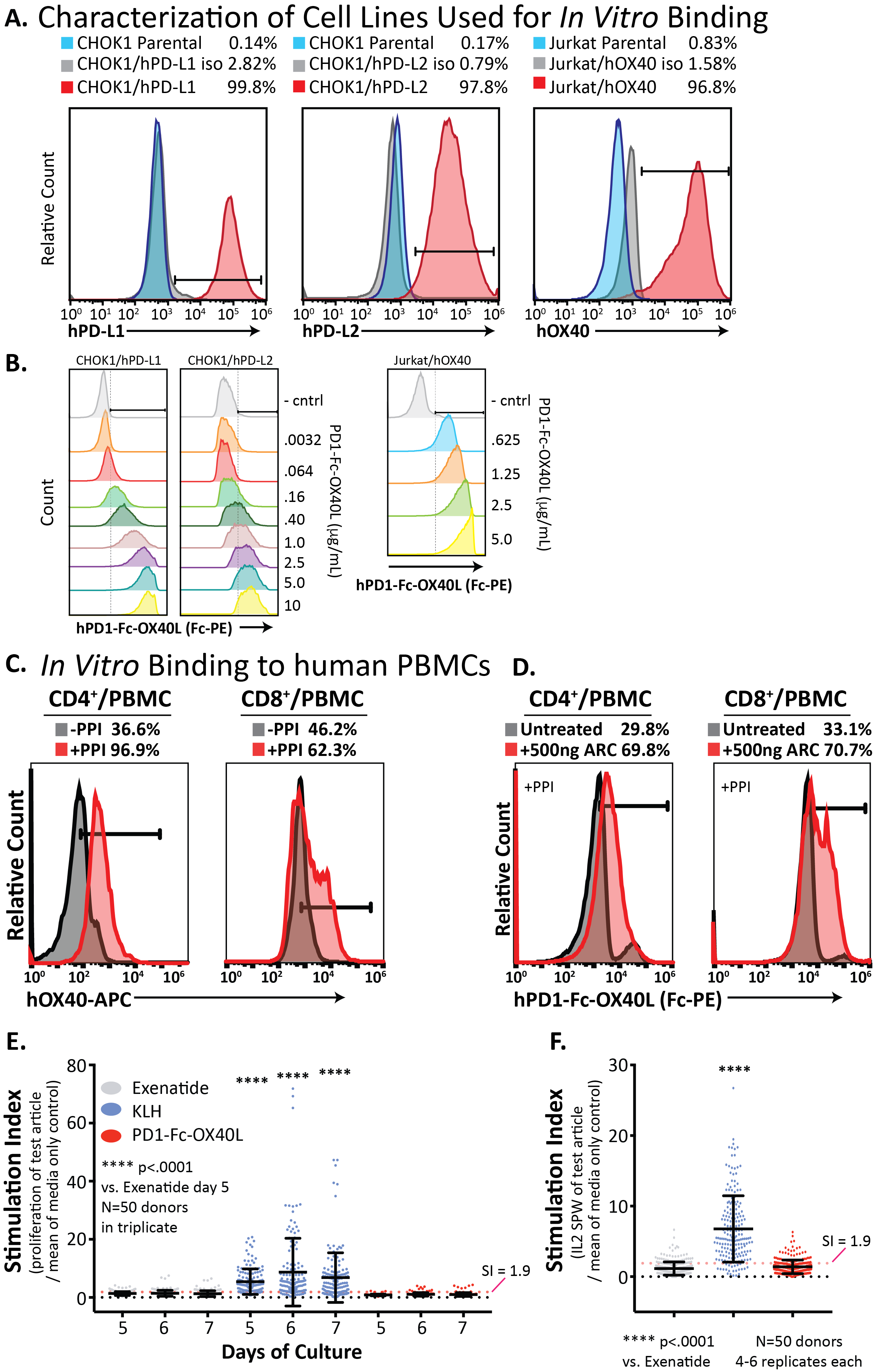

Supplement: Supplementary file 4 — Figure S3. Human in vitro cell line characterization and immunogenicity analysis. (TIF 1673 kb) [file 40425_2018_454_MOESM4_ESM.tif]

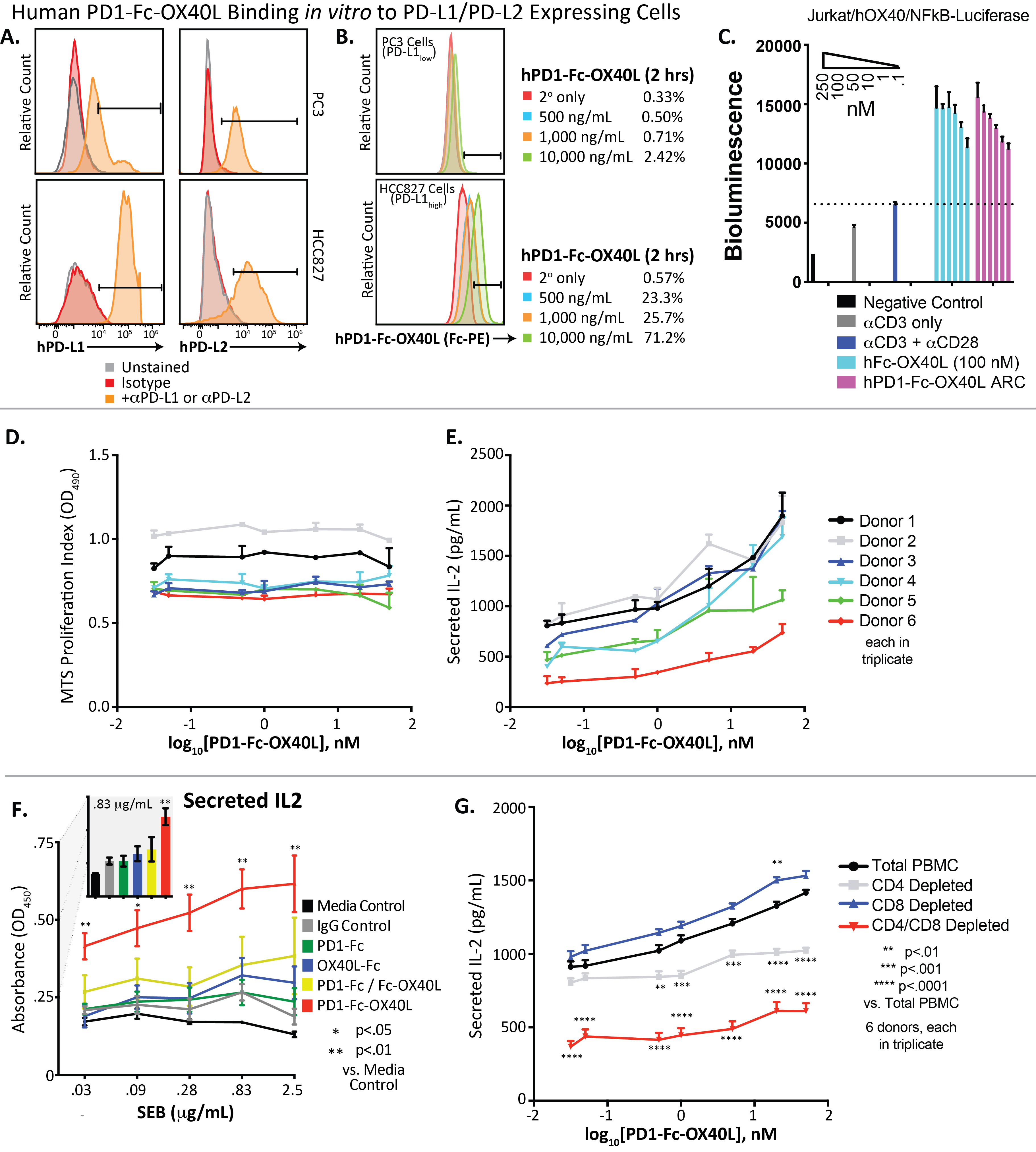

Supplement: Supplementary file 5 — Figure S4. Human tumor cell line characterization for PD-L1/L2 expression and PD1-Fc-OX40L ARC binding, SEB assay, and NFkB-luciferase reporter assay. (TIF 2037 kb) [file 40425_2018_454_MOESM5_ESM.tif]

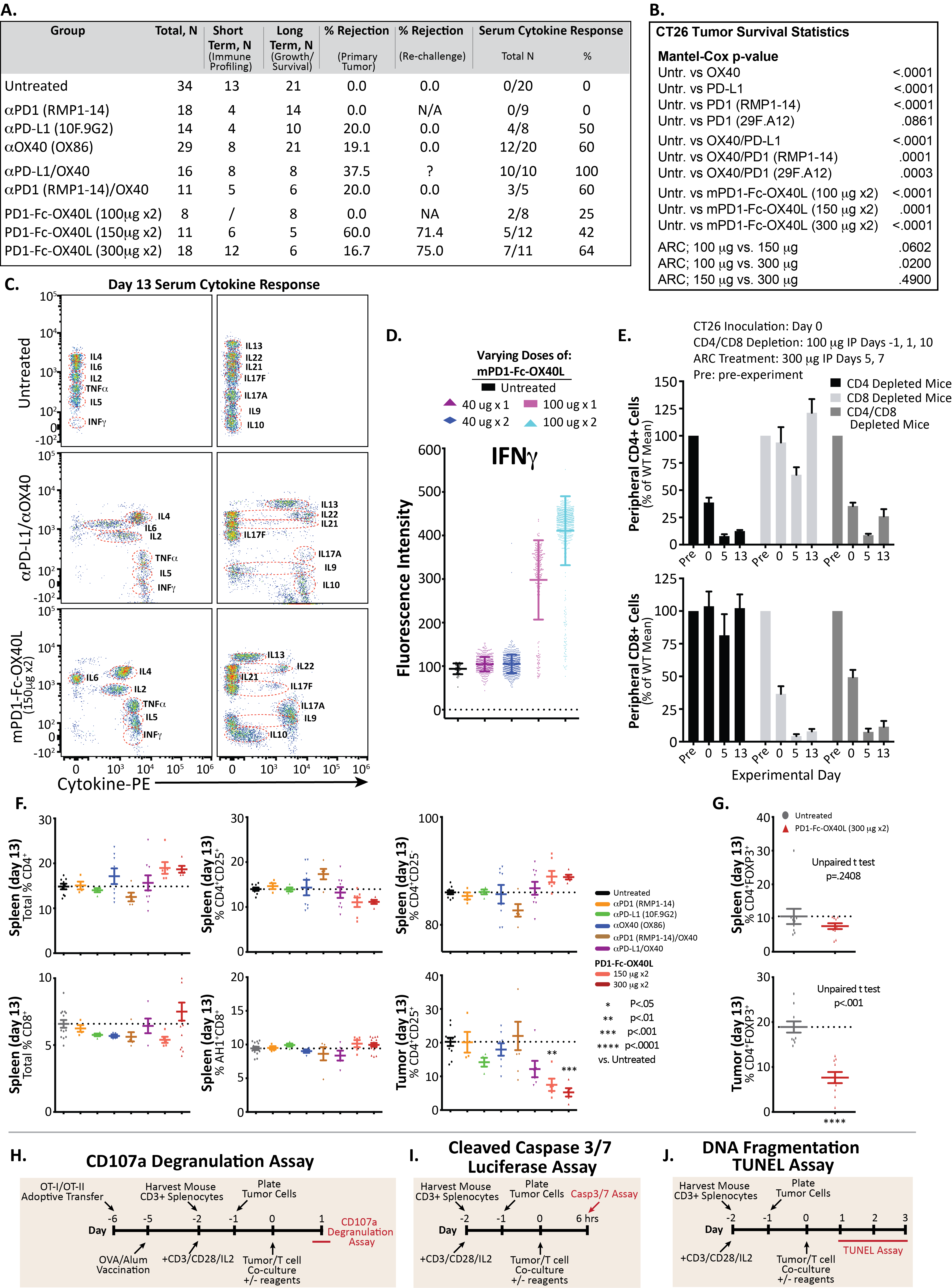

Supplement: Supplementary file 6 — Figure S6. Mouse PD1-Fc-OX40L efficacy ± CD4/CD8 depletion in CT26 tumor model and schematics of tumor killing/apoptosis assays performed in Fig. 6. (TIF 2696 kb) [file 40425_2018_454_MOESM6_ESM.tif]
